# Molecular evidence for convergent evolution and allopolyploid speciation within the *Physcomitrium*-*Physcomitrella* species complex

**DOI:** 10.1186/1471-2148-14-158

**Published:** 2014-07-11

**Authors:** Anna K Beike, Mark von Stackelberg, Mareike Schallenberg-Rüdinger, Sebastian T Hanke, Marie Follo, Dietmar Quandt, Stuart F McDaniel, Ralf Reski, Benito C Tan, Stefan A Rensing

**Affiliations:** 1 Faculty of Biology, University of Freiburg, Schänzlestr. 1, 79104 Freiburg, Germany; 2 Plant Biotechnology, Faculty of Biology, University of Freiburg, Schänzlestr. 1, 79104 Freiburg, Germany; 3 FRISYS Freiburg Initiative for Systems Biology, University of Freiburg, 79104 Freiburg, Germany; 4 Present address: Käthe-Kollwitz-Schule, Reserveallee 5, 76646 Bruchsal, Germany; 5 Nees Institute for Biodiversity of Plants, University of Bonn, Meckenheimer Allee 170, 53115 Bonn, Germany; 6 Department of Medicine I, University Medical Center Freiburg, 79106 Freiburg, Germany; 7 University of Florida, Gainesville, FL 32611, USA; 8 FRIAS Freiburg Institute for Advanced Studies, University of Freiburg, 79104 Freiburg, Germany; 9 BIOSS Centre for Biological Signalling Studies, University of Freiburg, 79104 Freiburg, Germany; 10 Plant Cell Biology, Faculty of Biology, University of Marburg, Karl-von-Frisch-Str. 8, 35043 Marburg, Germany; 11 The University and Jepson Herbaria, University of California, Berkeley, CA 94720, USA

**Keywords:** *Physcomitrella patens*, Funariaceae, Hybridization, Polyploidization, Speciation

## Abstract

**Background:**

The moss *Physcomitrella patens* (Hedw.) Bruch & Schimp. is an important experimental model system for evolutionary-developmental studies. In order to shed light on the evolutionary history of *Physcomitrella* and related species within the Funariaceae, we analyzed the natural genetic diversity of the *Physcomitrium*-*Physcomitrella* species complex.

**Results:**

Molecular analysis of the nuclear single copy gene *BRK1* reveals that three *Physcomitrium* species feature larger genome sizes than *Physcomitrella patens* and encode two expressed *BRK1* homeologs (polyploidization-derived paralogs), indicating that they may be allopolyploid hybrids. Phylogenetic analyses of *BRK1* as well as microsatellite simple sequence repeat (SSR) data confirm a polyphyletic origin for three *Physcomitrella* lineages. Differences in the conservation of mitochondrial editing sites further support hybridization and cryptic speciation within the *Physcomitrium*-*Physcomitrella* species complex.

**Conclusions:**

We propose a revised classification of the previously described four subspecies of *Physcomitrella patens* into three distinct species, namely *Physcomitrella patens*, *Physcomitrella readeri* and *Physcomitrella magdalenae*. We argue that secondary reduction of sporophyte complexity in these species is due to the establishment of an ecological niche, namely spores resting in mud and possible spore dispersal by migratory birds. Besides the *Physcomitrium*-*Physcomitrella* species complex, the Funariaceae are host to their type species, *Funaria hygrometrica*, featuring a sporophyte morphology which is more complex. Their considerable developmental variation among closely related lineages and remarkable trait evolution render the Funariaceae an interesting group for evolutionary and genetic research.

## Background

A major goal in evolutionary biology is to understand the processes that generate new species. Until recently, genetic analyses of species differences have relied on a small number of model systems. Here we examine patterns of divergence among relatives of the moss *Physcomitrella patens* (Hedw.) Bruch & Schimp., the first bryophyte with completely sequenced and well-annotated nuclear, plastid and mitochondrial genomes [1-4]. *P. patens* belongs to the Funariaceae, a family of small, terricolous mosses with highly diverse sporophyte morphology. In contrast to other Funariaceae, *Physcomitrella* is characterized by a short sporophyte lacking many of the ornamentations typical in mosses. The history of the genus was reviewed by Tan [5]. The Index Muscorum [6] listed four species in the genus *Physcomitrella*. Of the four taxa, *Physcomitrella austro-patens* Broth. and *P. californica* H.A. Crum and L.E. Anderson were treated later as synonyms of *P. readeri* (Müll. Hall.) I.G. Stone & G.A.M. Scott. *Physcomitrella hampei* Limpr. was interpreted as a hybrid species [5-7]. However, based on variable but overlapping phenotypic characteristics, a revised classification of the genus *Physcomitrella* was subsequently proposed by Tan [5], which described *Physcomitrella* as one single polymorphic species with four subspecies, namely *P. patens* ssp. *patens* from Europe, *P. patens* ssp. *readeri* (Müll. Hal.) B.C. Tan from Australia, *P. patens* ssp. *californica* (H.A. Crum & L.E. Anderson) B.C. Tan from California (North America) and Japan, and *P. patens* ssp. *magdalenae* (de Sloover) B.C. Tan from Rwanda (Africa). Currently, the majority of bryologists accept three separate species, namely *Physcomitrella patens*, *P. readeri* and *P. magdalenae* De Sloover. *P. patens* has a wide distribution in the Northern Hemisphere, *P. readeri* is found in California (North America), Australia and Japan [8], while *P. magdalenae* has been reported from Rwanda, Africa [9,10]. Recent data suggest that the *Physcomitrella* phenotype arose three times within the *Physcomitrium*-*Physcomitrella* species complex, based on phylogenetic analyses of nuclear, chloroplast, and mitochondrial DNA sequence data [11,12]. Here, the species complex is defined as a taxonomic group of intergraded phenotypes that hinders separation based on morphological traits. Due to the fact that *Physcomitrella* has been classified as a single species based on similar morphological characters of the sporophytes, it has been argued that such characters should not be used for classification [11]. In order to test the polyphyletic origin of the genus *Physcomitrella* and to analyze whether monophyletic groups corresponding to species can be resolved within *Physcomitrella*, we performed phylogenetic analyses of a nuclear single copy gene (*BRK1*) [13] and microsatellite simple sequence repeat (SSR) data amplified from numerous accessions covering all four *Physcomitrella* subspecies and further Funariaceae.

Regarding the sequenced *P. patens* strain from Gransden (Europe), the haploid chromosome number of n = 27 for meiotic and mitotic cells [14,15] provides evidence for a complex history of polyploidization, since the base number of chromosomes is reported to be n = 4–7 among mosses [16-18]. Genome duplication or polyploidization is an important mechanism of eukaryotic evolution [19-22] and considered to be of particular relevance in the speciation and diversification of land plants. Molecular data have confirmed that *P. patens* is a paleopolyploid that underwent at least one whole-genome duplication event approximately 45 MYA during the Eocene [23]. However, some other Funariaceae from within the *Physcomitrium*-*Physcomitrella* species complex have even higher chromosome numbers ranging, e.g., from n = 9 to n = 72 for *Physcomitrium pyriforme*, or n = 9 to n = 54 for *Physcomitrium eurystomum*[18]. Taking this into account, along with the fact that some Funariaceae show interfertility [5,12,24,25], a considerable number of polyploids, including allopolyploid hybrid species, can be expected. Natural hybrids among the Funariaceae, typically characterized by intermediate sporophytic characteristics [26,27], have also been described from the field [28-31]. The putative hybrid origin of *Physcomitrium collenchymatum* and *P. eurystomum* was recently been suggested based on molecular data and genealogical analyses of six different loci, including ribosomal, plastidic, and nuclear marker genes [12]. However, scarce evidence for polyploidization-derived paralogs (homeologs) of single copy genes in the analyzed *Physcomitrium* species has been shown to date.

In this study, we analyzed genome sizes and homeologs of the nuclear single copy gene *BRK1*[13] across a broad range of Funariaceae accessions in order to test whether species belonging to the genus *Physcomitrium* are allopolyploid hybrids. We chose *BRK1* as a phylogenetic marker gene as it is a single copy gene in nearly all of the land plant genomes sequenced to date (Additional file 1: Figure S2). In addition, we assessed the requirement of RNA editing sites to be edited, since out of 13 *P. patens* editing sites (cytidines which are post-transcriptionally changed into uridines) [32,33] three are not present in *Funaria hygrometrica*[34], thus rendering the pattern of editing site gain and loss a potentially informative evolutionary feature within the Funariaceae. Based on phylogenetic analysis of the novel marker gene *BRK1*, microsatellite-derived genetic distances and different editing patterns, we have revised the *Physcomitrella* subspecies *sensu* Tan [5] and hypothesize on speciation and the mode of spore dispersal in *Physcomitrella*.

## Methods

### Funariaceae *in vitro* collection, culture and observation

Numerous Funariaceae accessions (determined by the collectors) were contributed to the authors (Table 1) and established in axenic *in vitro* culture as previously described [12,35]. All plants originated from recent isolates except the *P. patens* accession from Gransden (Europe) which derives from a single spore isolated by H.L.K. Whitehouse in 1962 [14]. Information about locality, habitat, collectors, year of collection, International Moss Stock Center (IMSC) numbers and vouchers, as well as molecular, morphological and flow cytometric data are available for selected species from that collection (Additional file 2: Table S1). Plants from two capsules collected at the same location were denoted with a “K” and the capsule number. At present, the *in vitro* collection comprises 38 Funariaceae accessions from different worldwide locations (Additional file 2: Table S1). These accessions were assigned to taxa according to the species classification of the collectors and encompass 20 *Physcomitrella*, 14 *Physcomitrium*, three *Funaria* and one *Aphanorrhegma* accession. The *Physcomitrella* accessions comprise plants from regions where the four different subspecies were originally found [5,10]. *P. patens* ssp. *patens* is represented by 14 accessions from different regions of Europe and one accession from North America. *P. patens* ssp. *californica* accessions are available from North America and Japan, one from the southern island Kyushu and two from the central island Honshu. *P. patens* ssp. *magdalenae* was collected in Rwanda, Africa. One accession of *P. patens* ssp. *readeri* is available from the Melton Reservoir in Australia. In addition to that, *Aphanorrhegma serratum* and three *Funaria* species from North America are represented in the collection. Concerning *Physcomitrium*, three *P. sphaericum* accessions, three *P. eurystomum* accessions, seven *P. pyriforme* accessions from Europe and North America and one *P. collenchymatum* accession from North America are also available (Table 1, Additional file 2: Table S1).

**Table 1:** Funariaceae species collection

**Species (revised)**	**Locality**	**Region**	**Collector**	***Physcomitrella subspecies *** **(*sensu *** **Tan)**
*Physcomitrella patens*	Gransden Wood, Huntigdonshire, UK	Europe	Whitehouse	*patens*
	Nene Washes, Cambridge, UK	Europe	Preston	*patens*
	Cholsey, Berkshire, UK	Europe	Porley	*patens*
	Bad Honnef, Grafenwerth, Rheinland-Pfalz, Germany	Europe	Frahm	*patens*
	Heimerbrühl, Rheinland-Pfalz, Germany	Europe	Wolff	*patens*
	Nennig, Saarland, Germany	Europe	Caspari	*patens*
	Martinshof, Saarland, Germany	Europe	Caspari	*patens*
	Villersexel, Haute Saône, France (K3 + K4)	Europe	Lüth	*patens*
	Lviv, Ukraine	Europe	Lobachevska	*patens*
	Wik castle, Uppsala, Sweden	Europe	Nilsson, Thelander, Olsson, Ronne	*patens*
	Trondheim, Norway	Europe	Hassel	*patens*
	Gemünd, Nordrhein-Westfalen, Germany (K5)	Europe	Frahm	*patens*
	Gemünd, Nordrhein-Westfalen, Germany (K1) (var. megapolitana)	Europe	Frahm	*patens*
	Kaskaskia Island, Illinois, USA	North America	Sargent & Vitt	*patens*
	Del Valle Lake, California, USA	North America	Mishler	*californica*
*Physcomitrella readeri*	Kumamoto, Shisui-cho, Kyushu, Japan	Japan	Ono & Deguchi	*californica*
	Saitama, Iwatsuki-shi, Honshu, Japan	Japan	Higuchi	*californica*
	Okayama, Honshu, Japan	Japan	Hasebe	*californica*
	Melton Reservoir, Victoria, Australia	Australia	Stajsic & Klazenga	*readeri*
*Physcomitrella magdalenae*	Mt. Bisoke, Ruhengeri, Rwanda	Africa	Solga & Buchbender	*magdalenae*
*Physcomitrium sphaericum*	Grosshartmannsdorf, Osterzgebirge, Sachsen, Germany	Europe	Frahm	
	Imsbach-Aue, Saarland, Germany	Europe	Wolff	
	Vellescot, Territore-de-Belfort, France	Europe	Lüth	
*Physcomitrium eurystomum*	Neukirch, Allgäu West, Wangen, Bodenseekreis, Baden-Württemberg, Germany	Europe	Schäfer-Verwimp	
	Neustadt, Thüringen, Germany	Europe	Eckstein	
	Schleiz, Thüringen, Germany	Europe	Eckstein	
*Physcomitrium pyriforme*	Bischofswerda, Sachsen, Germany	Europe	Eckstein	
	Nordhausen, Liebenrode, Thüringen, Germany	Europe	Eckstein	
	Waltershof, Gera, Thüringen, Germany	Europe	Eckstein	
	Haardtrand, Ebekoben, Rheinland-Pfalz, Germany	Europe	Lauer	
	Övergran, Biskops-Arnö, Uppland, Sweden	Europe	Lönnell	
	Madeira, Portugal	Europe	Eckstein	
	Durham, Orange County, North Carolina, USA	North America	Goffinet	
*Physcomitrium collenchymatum*	Shaw Nature Reserve, Franklin County, MO, USA (K1, K2A, K2B)	North America	Allen & Darigo	
*Funaria hygrometrica*	Durham, Orange County, North Carolina, USA	North America	Goffinet	
*Funaria americana*	Canada, Alberta	North America	Goffinet	
*Funaria flavicans*	Durham, Orange County, North Carolina, USA	North America	Goffinet	
*Aphanorrhegma serratum*	Arkansas, USA	North America	Buck	

Several Funariaceae were contributed to the authors and established in axenic *in vitro* culture. The name of each species, locality, region and collector are listed for each accession. For *Physcomitrella*, the revised taxon name according to the new classification proposed here is listed on the left, while the former name of the subspecies as described by the collector is listed on the right. For further information, e.g. year of collection, habitat and available data (*BRK1*, SSRs, editing analysis, genome size, IMSC number) see Additional file 2: Table S1.

Plants were cultivated on solid mineral Knop medium [36] as previously described [37]. For standardized observation of gametophytic features, plants were grown on Petri dishes (9 cm diameter) wrapped with laboratory film. Environmental conditions were set to 22 degrees Celsius and a long day (16 h light, 8 h dark) light cycle (white light at 70 μmol * s^-1^ * m^-2^). Plants were established by transfer of individual gametophores, and observed under a stereo binocular (Zeiss, Germany) after several weeks to months of growth.

### Genomic DNA extraction

Genomic DNA was extracted from moss tissue following the cetyltrimethyl ammonium bromide (CTAB) method described by [38]. Up to 100 mg of moss material was disrupted with a Tissue Lyser (Qiagen, Hilden, Germany) and incubated for 30 min in 700 μL CTAB buffer (2 % CTAB, 1.4 M NaCl, 20 mM EDTA, 0.5 % PVP 40, 100 mM Tris/HCl, pH 8.0; 0.2 % 2-mercaptoethanol [v/v] added before use). Subsequently, 600 μL chloroform:isoamylalcohol (24:1) was added. Phase separation was reached after vigorous mixing by centrifugation (16,100 × g) for 5 min. The aqueous phase was transferred to a fresh tube and 2/3 [v:v] isopropanol was added for precipitation at -20 °C overnight. The DNA was sedimented by centrifugation (20,817 x g) for 30–45 min at 4 °C. The supernatant was removed and the pellet was washed twice with 200 μL of 70 % ethanol. After centrifugation (16,100 x g), the supernatant was removed. DNA was dissolved in 100 μL TE buffer (0.1 M TrisHCl, 0.01 M EDTA, pH 7.5 with HCl). For RNAse digestion, 10 μg RNAse A (10 mg/mL, Thermo Scientific, St. Leon-Rot, Germany) were added and the DNA was incubated for 1 h at 37 °C. RNAse digestion was controlled and DNA concentration was determined by gel electrophoresis in 1 % agarose gels.

### Amplification of *BRK1* from genomic DNA

A part of the nuclear gene *BRK1* (Pp1s35_157V6.1) was amplified using the primers BRICK1_for: GTCGGCATTGCTGTACAA and BRICK1_rev: CTCCAGCTGACGCTCCAG. The PCR was performed in 20 μL reaction volume containing 2 μL 10 × Buffer E (Genaxxon, Biberach, Germany), 0.4 μL deoxyribonucleotide triphosphates (dNTPs, 10 mM, Thermo Scientific, St. Leon-Rot, Germany), 1.25 U Taq polymerase (Genaxxon, Biberach), 0.5 μl of each primer (10 pmol/μL) and 1 μL genomic DNA (50–100 ng/μL). The PCR cycling conditions consisted of an initial denaturation at 94 °C for 5 min, followed by cycling conditions which consisted of a denaturation step of 45 s at 94 °C, annealing at 52 °C for 1 min and elongation at 72 °C for 1 min for a total of 30 cycles. The PCR products were eluted and purified from a 1 % agarose gel with the QIAquick Gel Extraction Kit (Qiagen, Hilden, Germany) according to the manufacturer’s instructions.

### Cloning of *BRK1* and plasmid DNA extraction

**Figure 1:**
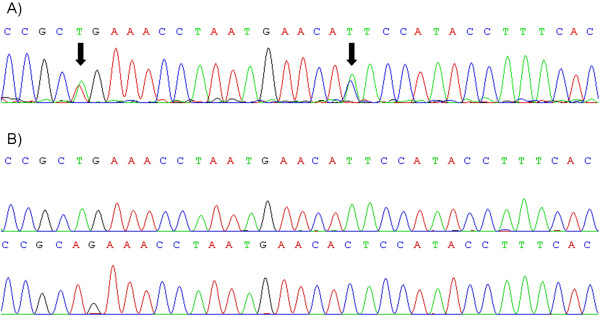
**Electropherograms of the *BRK1 *intron region from *Physcomitrium eurystomum. *(A)** Direct sequencing product of *BRK1* amplified from *P. eurystomum* (Schleiz, Europe) genomic DNA. Sequence polymorphisms are indicated with black arrows. **(B)** Clonal sequencing products of the *BRK1* showing two distinct homeologs of *BRK1* in *P. eurystomum*. Figure generated using Gentle (http://gentle.magnusmanske.de/).

The PCR sequencing products of *P. eurystomum*, *P. collenchymatum* and *P. pyriforme* showed overlapping sequence peaks (polymorphisms) in the electropherogram (Figure 1A). In order to obtain clear sequences for potentially multiple loci of *BRK1* for these species, the PCR products were cloned into the blunt end vector pJET1.2/blunt according to the manufacturer’s protocol (CloneJET™ Molecular Cloning Kit, Thermo Scientific, St. Leon-Rot, Germany). The vector was transformed into *E. coli* cells, which were incubated overnight on solid ampicillin-containing LB medium at 37 °C for selection. Bacterial colonies were screened for the insertion of plasmid DNA via PCR using the pJET1.2/blunt primers pJet_T7_for: TAATACGACTCACTATAGGG and pJet_rev: GAAGAACATCGATTTTCCATGGCAGC. Plasmid DNA extraction was performed after an overnight incubation in ampicillin-containing liquid LB medium at 37 °C using the GeneJET™ Plasmid Miniprep-Kit (Thermo Scientific, St. Leon-Rot, Germany).

### Direct sequencing of *BRK1* PCR products and of plasmid clones

In total, 10–50 ng/μL of the purified PCR product were sent for sequencing to GATC Biotech AG (Konstanz, Germany) using the primers BRICK1_for and BRICK1_rev. Plasmid DNA was sent in concentrations of 30–100 ng/μL and Sanger sequenced (GATC Biotech AG, Konstanz, Germany) with a standard sequencing primer for the blunt end vector pJET1.2/blunt (Thermo Scientific, St. Leon-Rot, Germany). For accessions with polymorphic direct PCR sequencing products, up to 10 plasmid DNAs from independent clones were sent for sequencing, respectively. Sequences which were obtained at least twice independently were considered true and used for phylogenetic analyses. Exceptions to this were both sequences of *BRK1* for one *P. pyriforme* accession (Nordhausen, Europe), and one sequence representing one locus of *P. collenchymatum* (Shaw Nature Reserve, Franklin County, MO, USA) which were each obtained only once. These sequences were included into further analyses since they were found to be identical to corresponding sequences of other accessions from the same species.

### *BRK1* sequence analysis

The sequence chromatograms were analyzed with the ChromasPro software, version 1.34 (http://www.technelysium.com.au/ChromasPro.html). Multiple sequence alignments were calculated with MUSCLE 3.51 [39] and visualized with Jalview [40]. The gene structure of *BRK1* was annotated within the multiple sequence alignment according to the gene structure found in *P. patens* (Pp1s35_157V6.1, Additional file 3: Figure S1). Neighbor-joining analysis was performed with QUICKTREE_SD [41,42] using 1,000 bootstrap replicates. Bayesian inference was carried out with MrBayes [43], using the GTR model with eight gamma distributed rates, invariant sites, two hot and two cold chains, burn-in 250 and two million generations, until the standard deviation of split frequencies dropped below 0.01. Maximum likelihood inference was carried out with TREE-PUZZLE [44] using the GTR model with eight gamma-distributed rates, quartet puzzling (10,000 steps) and exact parameter estimation. While the Maximum Likelihood tree is shown, support values from all three methods are plotted on the tree (see legend). The tree was visualized with FigTree v1.1.2 (http://tree.bio.ed.ac.uk/software/figtree/).

### RNA editing analysis

DNA was prepared from plant material using the extraction method described by [45]. Primer pairs bordering regions of *nad*3, *nad*4, *nad*5 (subunits of complex I, amplicon length 343 bp, 392 bp and 459 bp, respectively), *cox*1 (subunit of complex IV, 252 bp), *rps*14 (ribosomal protein S14, 246 bp) and *ccm*FC (cytochrome biogenesis factor subunit C, 210 bp) harboring mitochondrial editing sites identified in the *P. patens* accession from Gransden [34], were used for PCR assays. Amplification assays were performed as described [34]. PCR products were gel-purified using the HiYield PCR Clean-up & Gel-Extraction kit (Südlaborbedarf GmbH, Gauting, Germany) or purified using ExoSAP-IT (Affymetrix, Santa Clara, USA) and Sanger sequenced (GATC Biotech AG, Konstanz, Germany). DNA sequences were aligned to the corresponding coding sequences of *P. patens* and *Funaria hygrometrica*[34] using Mega 5.0 [46] and putative editing sites were identified via PREPACT 2.0 [47].

### Amplification of EST-derived microsatellites

Genomic DNA was extracted as previously described [48]. Sixty-four simple sequence repeat (SSR) loci with available polymorphism information content (Table http://www.cosmoss.org/376_PCR_tested_SSRs.xls on http://www.cosmoss.org/genmap.content) were chosen from a collection of EST-derived microsatellites [48]. In total, 49 loci were found to be suitable by means of PCR, using the respective first primer pair listed; the SSR numbering in Additional file 4: Table S2 corresponds to the one in the above mentioned original data. SSR loci were amplified in a 20 μL PCR mix containing 2 μl of 10 × RED-Taq-PCR buffer, 0.1 mM dATP, dCTP, dGTP and dTTP, 5 pmol each of two primers, 0.5 U RED-Taq-Polymerase (Sigma-Aldrich, Deisenhofen, Germany) and 4 ng genomic DNA. PCR was carried out starting with an initial DNA denaturation at 95 °C for 2 min. The first cycle consisted of 30 s denaturation at 92 °C, with primer annealing for 30 s at 60 °C and elongation for 30 s at 72 °C. In each of the 10 subsequent cycles, the annealing temperature was decreased by 0.7 °C. The final 25 cycles consisted of 15 s denaturation at 92 °C, 15 s primer annealing at 52 °C and 30 s elongation at 72 °C. SSR PCR products were size separated in 3 % MetaPhor (Cambrex Corporation, East Rutherford, USA) high resolution agarose by gel electrophoresis in 0.5 fold TBE (45 mM Tris-borate, 1 mM EDTA, pH 8.0) and visualized by ethidium bromide staining.

### SSR data analysis

SSR loci were scored manually for all included accessions according to their amplified fragment size. Distinguishable sizes were scored as different alleles; indistinguishable sizes were scored as the same allele (Additional file 4: Table S2). Absence of PCR products was scored as a haploid null allele. The data set included all *Physcomitrella* accessions as well as *Physcomitrium sphaericum* (Additional file 2: Table S1). Genetic distances were calculated with Nei’s DA distance algorithm [49]. The phylogenetic tree was constructed with the Neighbor-Joining algorithm [50] using 1,000 bootstrap replicates. For genetic distance and phylogenetic tree calculations the software POPULATION 1.2.28 [51] was used and the tree was visualized using FigTree (http://tree.bio.ed.ac.uk/software/figtree/). In addition, the different sized alleles derived from the 49 SSR loci were transformed into 170 binary presence/absence characters (null alleles were scored as gaps). The binary encoding might overestimate variation, but was necessary since SplitsTree does not allow presentation of the data in a continuous fashion. Based on this matrix (Additional file 4: Table S2), SplitsTree 4 [52] was used to calculate GeneContent distances and a bootstrapped (1,000 replicates) NeighborNet.

### Flow cytometry

Gametophores, pre-cultured for four weeks, were chopped using a razor blade in buffer (0.107 g MgCl_2_* 6 H_2_O, 0.5 g NaCl, 1.211 g Tris, 0.1 mL Triton X-100 in 100 mL water, pH 7.0 with HCl) containing 1 mg/L 4.6 Diamidino-2-Phenylindol (DAPI). The debris was filtered through a 40 μm sieve prior to measurement. The flow cytometric measurement was performed with a MoFlo High Speed Cell Sorter (Beckman Coulter, Krefeld, Germany). The nuclei were sorted on the basis of their forward and side scatter profiles as well as on their DAPI signals. The scatter measurements were performed using excitation from an argon ion laser at 488 nm at 200 mW power, while the DAPI signal came from excitation with an argon ion laser set up for multiline UV emission (351–356 nm) at 80 mW. DAPI emission was measured from 440 to 460 nm. As a reference to control for the relative intensities of the samples, 20 μL of DAPI Reference Standard Beads (Bangs Laboratories, Inc., Cat. 906, Lot-No. 7238) were added to each sample. The DAPI beads could be separated from the nuclei on the basis of their forward versus side scatter profiles. Profiles were separated into DAPI, 1n (1c) and 2n (2c) peaks. The 2n peak represents cells arrested at the G2/M transition [53]. Values presented in Table 2 are the averaged median of the 1n peak divided by the median of the peak for the DAPI beads, i.e., haploid nuclear DNA content normalized to DAPI control beads. The ratio between the 1n and 2n peak was 1.99 +/- 0.19 in all samples, supporting correct peak definition (Additional file 5: Table S3). *P. patens* from Gransden served as an additional control for each experiment [n = 6]; other accessions were measured up to four times if in doubt of measurement quality.

**Table 2:** Genome sizes of Funariaceae

**Species (revised)**	**Genome size [1c]**	***Physcomitrella *** **subspecies (*sensu *** **Tan)**
*Physcomitrella patens*°	0.96 ± 0.15	*patens, californica*
*Physcomitrella readeri*	0.96 ± 0.05	*readeri, californica*
*Physcomitrella magdalenae*	0.92	*magdalenae*
*Physcomitrium sphaericum*	0.78 ± 0.43	
*Physcomitrium eurystomum*	1.27 ± 0.32*	
*Physcomitrium collenchymatum*	1.51 ± 0.44	
*Physcomitrium pyriforme*	1.33 ± 0.42*	
*Funaria hygrometrica*	0.44 ± 0.03*	
*Aphanorrhegma serratum*	0.9	

Genome size shown is the haploid (1c) peak normalized by DAPI beads (see Methods); values are shown +/- standard deviation where applicable. Asterisks show significantly different genome size with regard to *P. patens* (one-sided *t*-test, p < 0.05). °genome size for Gransden = 1.02 +/- 0.14, Villersexel “K3” = 0.87, Del Valle Lake (*californica sensu* Tan) = 0.75; for further details see Additional file 5: Table S3. For *Physcomitrella*, the revised taxon name according to the new classification proposed here is listed on the left, while the former name of the subspecies is listed on the right.

### Sequencing of *BRK1* transcripts from six selected Funariaceae

Total RNA was extracted using the Qiagen RNeasy Plant Mini Kit (Qiagen, Hilden, Germany) according to the manufacturer’s instructions, followed by on-column DNAse digestion. The resulting RNA samples from *P. patens* from Gransden (Europe), three accessions from *P. eurystomum* (Neukirch, Neustadt and Schleiz, Germany) and two accessions from *P. collenchymatum* (Shaw Nature Reserve, Franklin County, MO, USA) were reverse transcribed using Superscript III (Invitrogen™, Karlsruhe, Germany). The reverse primer BRK1_rev (CACCGTTAGCTTCTCGTTCA) was used for priming the first strand synthesis. The resulting cDNA was then further amplified using both *BRK1* primers, BRK1_for (GACAATCGCCATTTTTCGAG) and BRK1_rev, and subsequently Sanger sequenced (GATC Biotech AG, Konstanz, Germany). The primers used were selected based on multiple sequence alignment of clonal sequences.

### Quantitative Real-Time PCR and high-resolution-melting

Total RNA was extracted and cDNA was synthesized as described above. cDNA synthesis was verified by PCR. Primers for quantitative Real-Time PCR (qPCR) were designed to specifically amplify a 129 bp long fragment of the exon containing polymorphisms in *P. collenchymatum* (BRK1_for: GACAATCGCCATTTTTCGAG and BRK1_rev CACCGTTAGCTTCTCGTTCA). Each qPCR was carried out using the SensiMix HRM kit (Bioline, Lickenwalde, Germany) on a LightCycler480 (Roche, Mannheim, Germany) with the following parameters: 95 °C for 10 min, followed by 50 cycles of 95 °C, 60 °C and 72 °C, for 15 s each. High resolution melting analysis was subsequently carried out from 65 °C to 95 °C with a ramp rate of 0.02 °C/s. Melting curve data were analyzed using the Gene Scanning software (Roche, Mannheim, Germany) and a sensitivity setting of 0.4 with auto-grouping. Pre-melt and post-melt temperatures were chosen in the range of 77 °C and 84 °C respectively.

## Results

### Paralogs of the single copy gene *BRK1* and enlarged genome sizes provide evidence for allopolyploid hybrid *Physcomitrium* species

**Figure 2:**
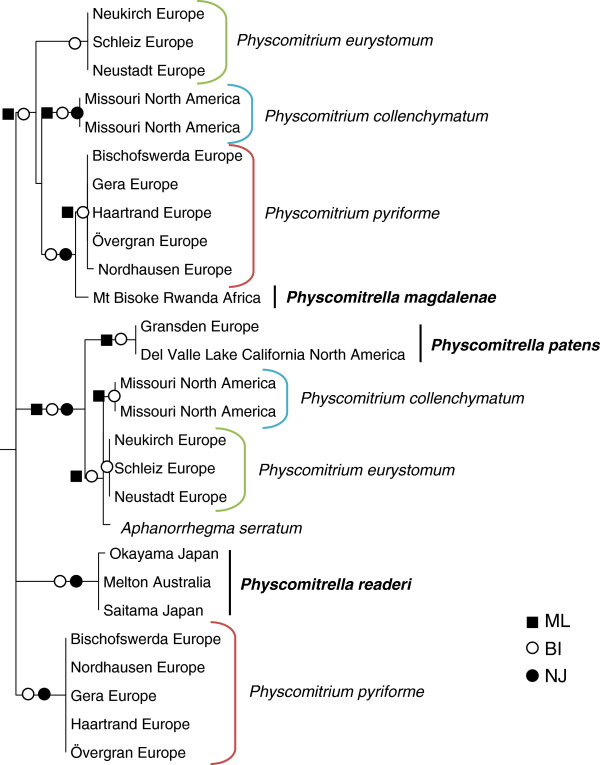
**Phylogenetic tree of *BRK1.*** Phylogenetic tree of the nuclear gene *BRK1* (Pp1s35_157V6.1) from selected Funariaceae. Three *Funaria* species (*F. hygrometrica*, *F. flavicans*, and *F. americana*) were used as the outgroup (not shown, see Additional file 6: Figure S3, for details). The cluster representing the *Physcomitrium*-*Physcomitrella* species complex shown here is supported by all methods. Due to differing or unresolved branching order, depending on inference method used, the backbone is shown as multifurcating. The three distinct clades of *Physcomitrella* representing *P. readeri* (Japan, Australia), *P. magdalenae* (Africa) and *P. patens* (Europe and North America) are shown in bold. The distinct paralogous loci of *BRK1* for each hybrid species are highlighted with colored brackets. Red brackets represent distinct loci of five *P. pyriforme* accessions from Europe. Blue brackets show multiple loci of two capsules of a *P. collenchymatum* accession from North America. Green brackets mark distinct loci of *BRK1* within three accessions of *P. eurystomum* from Europe. The symbols at the nodes represent support values > 95 for Maximum Likelihood (ML), Bayesian Inference (BI) and Neighbor Joining (NJ), respectively. They are derived from 1,000 NJ bootstrap samples, respectively show posterior probabilities of Bayesian inference or maximum likelihood quartet puzzling support.

In order to gain evidence for homeologs of the single copy gene *BRK1* we amplified this gene from selected Funariaceae, including the genera *Aphanorrhegma*, *Physcomitrella*, *Physcomitrium* and *Funaria* (Table 1, Additional file 2: Table S1). The sequence length amplified from genomic DNA (including the single intron) was approximately 600 nucleotides, but varies between the analyzed species (Table 3). In the PCR products, sequence polymorphisms were detected within all accessions of the species *P. pyriforme*, *P. collenchymatum* and *P. eurystomum*, indicating multiple *BRK1* loci within these species. However, no sequence polymorphisms were detected in the *Aphanorrhegma*, *Funaria* and *Physcomitrella* accessions (Table 3). After cloning the respective PCR products, two distinct sequences were clearly distinguishable per *Physcomitrium* species, respectively (Figure 1A). These loci were compared to the polymorphic direct sequencing product in order to compare the sequence polymorphisms with both clonal sequences of *BRK1* (Figure 1B). Most differences between the homeologs occurred in the single intron, while in the case of *P. collenchymatum* and *P. eurystomum* polymorphisms also occurred in the exons (Additional file 3: Figure S1, Table 3). Phylogenetic analyses revealed two distinct clades of the homeologous *BRK1* for *P. pyriforme*, *P. eurystomum* and *P. collenchymatum* (Figure 2). Since three of these clades (one per *Physcomitrium* lineage) cluster with the *P. patens* (ssp. *magdalenae*) accession from Africa (labelled *P. magdalenae* in Figure 2), and two of the other three with *P. patens* from Europe, they provide evidence for allopolyploidization rather than autopolyploidization.

**Table 3:** **Sequence polymorphisms of *BRK1***

**Species (revised)**	***BRK1 *** **sequence length (nucleotides)**	**Polymorphisms (exon/intron); gaps**
*Physcomitrella patens*	581	0
*Physcomitrella readeri*	580	0
*Physcomitrella magdalenae*	577	0
*Physcomitrium eurystomum*	614	27 (1/26); 1
*Physcomitrium collenchymatum*	614	26 (2/24); 1
*Physcomitrium pyriforme*	615	19 (0/19); 5
*Funaria flavicans*	580	0
*Funaria hygrometrica*	546	0
*Funaria americana*	580	0
*Aphanorrhegma serratum*	548	0

Overview of sequence polymorphisms in the direct PCR product of *BRK1* from different Funariaceae. For each species multiple accessions are included. The number of sequence polymorphisms and gaps between two homeologs from the *Physcomitrium* accessions is shown on the right. For details see Additional file 3: Figure S1.

Flow cytometric measurements were performed for all Funariaceae for which the *BRK1* sequence was available, including multiple accessions of *Physcomitrella*, *P. pyriforme*, *P. eurystomum* and *P. collenchymatum* (Additional file 2: Table S1). All accessions of *Physcomitrella* show a comparable genome size as *P. patens* from Gransden (Europe), whereas *P. pyriforme*, *P. eurystomum* and *P. collenchymatum* have a larger size (Table 2), albeit *P. collenchymatum* not significantly (p > 0.05, one-sided *t*-test). These higher amounts of DNA, together with the *BRK1* homeologs, indicate that *P. pyriforme*, *P. collenchymatum* and *P. eurystomum* are recent allopolyploids.

### Expression patterns of *BRK1* homeologs in *Physcomitrium* species

**Figure 3:**
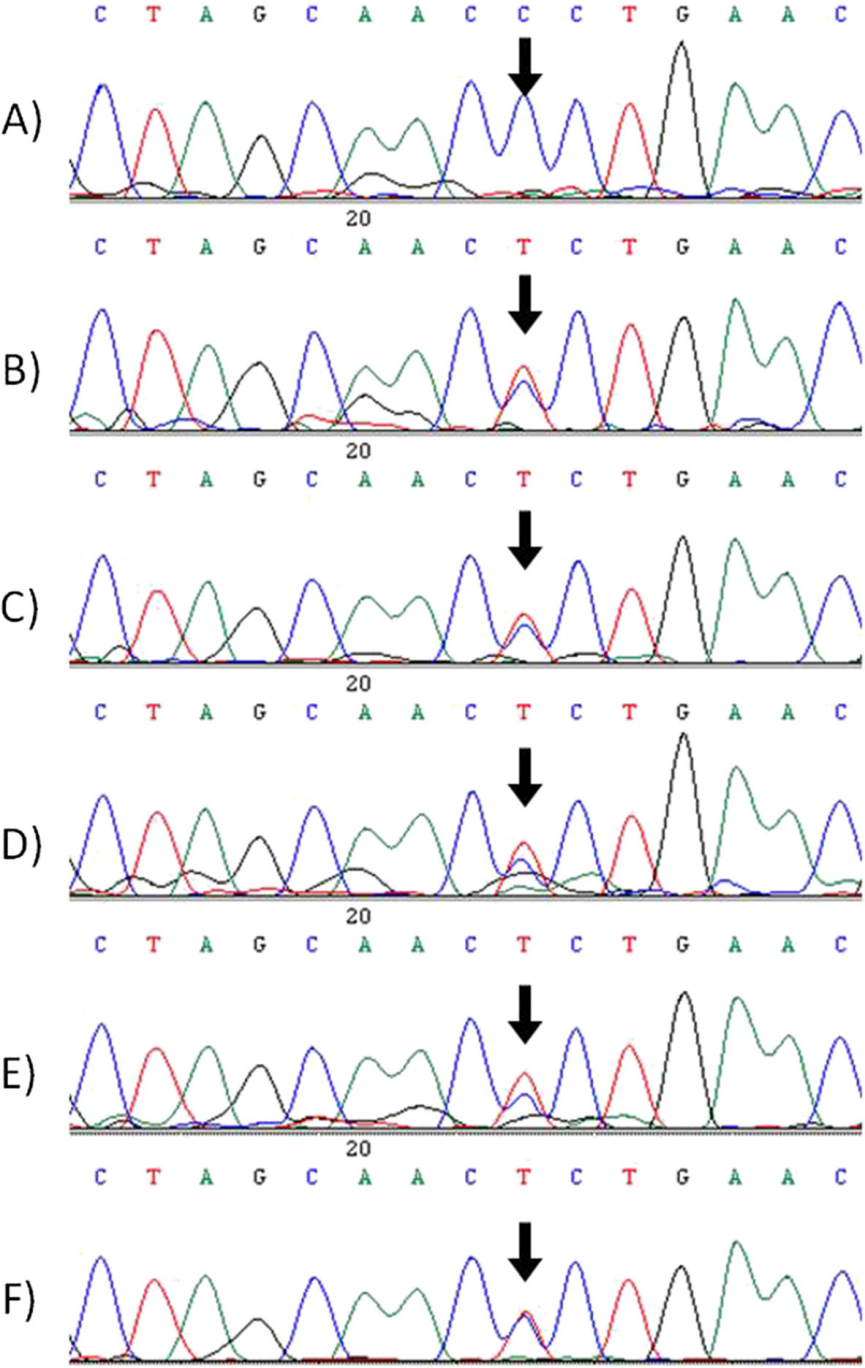
**Electropherograms of a polymorphic region within the *BRK1 *transcript from six selected Funariaceae.** Sequencing of an amplified region within the *BRK1* transcript from **(A)***P. patens* (Gransden*,* Europe)*, P. eurystomum* (Schleiz **(B)**, Neukirch **(D)** and Neustadt **(F)**, Europe) and *P. collenchymatum* (Shaw Nature Reserve, Franklin County, MO, USA, K1 **(C)** and K2A **(E)**, USA). Black arrows indicate sequence polymorphisms.

**Figure 4:**
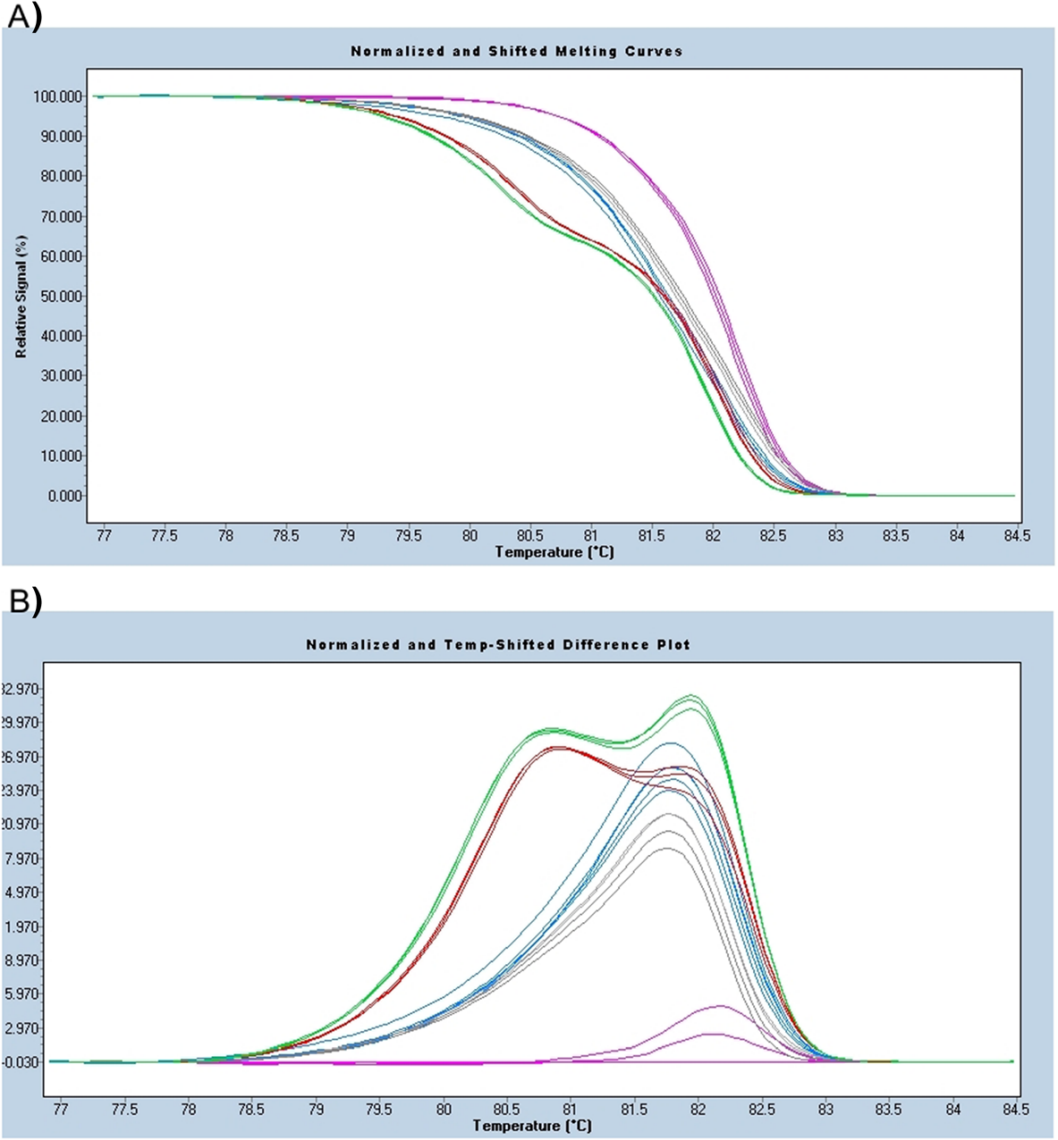
**High resolution melting analysis of *BRK1*. (A)** Normalized and temperature shifted high resolution melting curve and **(B)** difference plot of *BRK1* from *P. patens* (mauve), the two *P. collenchymatum* accessions K1 (green) and K2A (red) and the three *P. eurystomum* accessions Neustadt (gray) and Neukirch/Schleiz (both blue); see Additional file 3: Figure S1 for alignment with marked polymorphisms.

In order to determine whether both loci of *BRK1* are expressed in *P. collenchymatum* and *P. eurystomum*, RT-PCR and quantitative Real-Time PCR (qPCR) were performed using primers that cover polymorphisms in the exon (Additional file 3: Figure S1). The existence of both homeologs at the transcript level was confirmed via RT-PCR and subsequent sequencing of the transcripts (Figure 3). The expression pattern of the homeologs was determined with qPCR and the resulting amplicons were analyzed by high resolution melting (HRM) to distinguish the transcripts. *P. patens* from Gransden (Europe) was used as a reference. Both *P. collenchymatum* accessions show two distinct expressed transcripts of *BRK1*, whereas all three *P. eurystomum* and *Physcomitrella* yielded only one product in this analysis (Figure 4). Due to the almost identical melting patterns, the auto-grouping of the gene scanning software was not able to distinguish between the *P. eurystomum* accessions (Neukirch and Schleiz, Germany) with their single polymorphism at the exon level, while the two polymorphisms in the exon of *P. collenchymatum* (Table 3) could be resolved. In summary, the exon polymorphisms of *BRK1* were confirmed at the transcript level by sequencing (Figure 3) and partly by HRM, demonstrating that both homeologs are expressed (Figure 4).

### *BRK1* as a phylogenetic marker confirms polyphyletic origin of three *Physcomitrella* species

Based on the high conservation of BRK1 (Additional file 1: Figure S2) and on the fact that the encoding gene *BRK1* harbors an intron, we expected the nucleic acid sequence to represent a suitable phylogenetic marker to resolve relationships within the Funariaceae. Different methods of tree inference based on the nucleic acid sequences (exon/intron) all led to essentially the same topology, separating the *Funaria* sequences from four well supported clades (Additional file 6: Figure S3). The three clades representing *Physcomitrella* from Europe and North America (ssp. *patens* and ssp. *californica*), *Physcomitrella* from Australia and Japan (ssp. *readeri* and ssp. *californica*) and *P. patens* ssp. *magdalenae* from Africa are distinct in the *BRK1*-based phylogeny (Figure 2, Additional file 6: Figure S3). This confirms a polyphyletic origin of these three lineages as previously proposed [11,12] and supports that accessions of *P. patens* ssp. *californica* (Del Valle Lake, California and two accessions from Japan) actually belong to two different clades. Moreover, *A. serratum* clusters in a clade together with *P. patens* ssp. *patens* and may therefore be considered to belong to the *Physcomitrium*-*Physcomitrella* complex as well.

### Genic microsatellites support high genetic distances within the genus *Physcomitrella*

**Figure 5:**
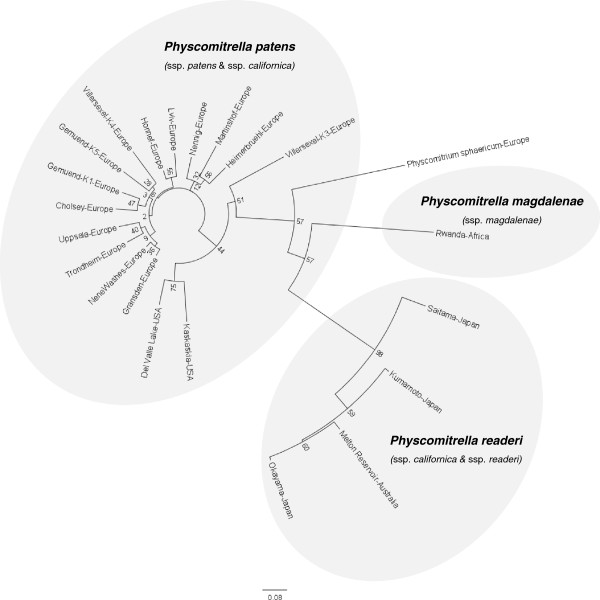
**SSR-based neighbor-joining tree.** Unrooted neighbor-joining tree constructed using Nei’s DA-distance on datasets derived from 64 SSRs of 21 *Physcomitrella* accessions. Accessions are named with their designation and country. Gray ellipses indicate three clades of *Physcomitrella* accessions, supporting a revised classification into the three distinct species *P. patens* (Europe and North America), *P. readeri* (Australia and Japan) and *P. magdalenae* (Africa). The numbers at the nodes are derived from 1,000 bootstrap samples.

**Figure 6:**
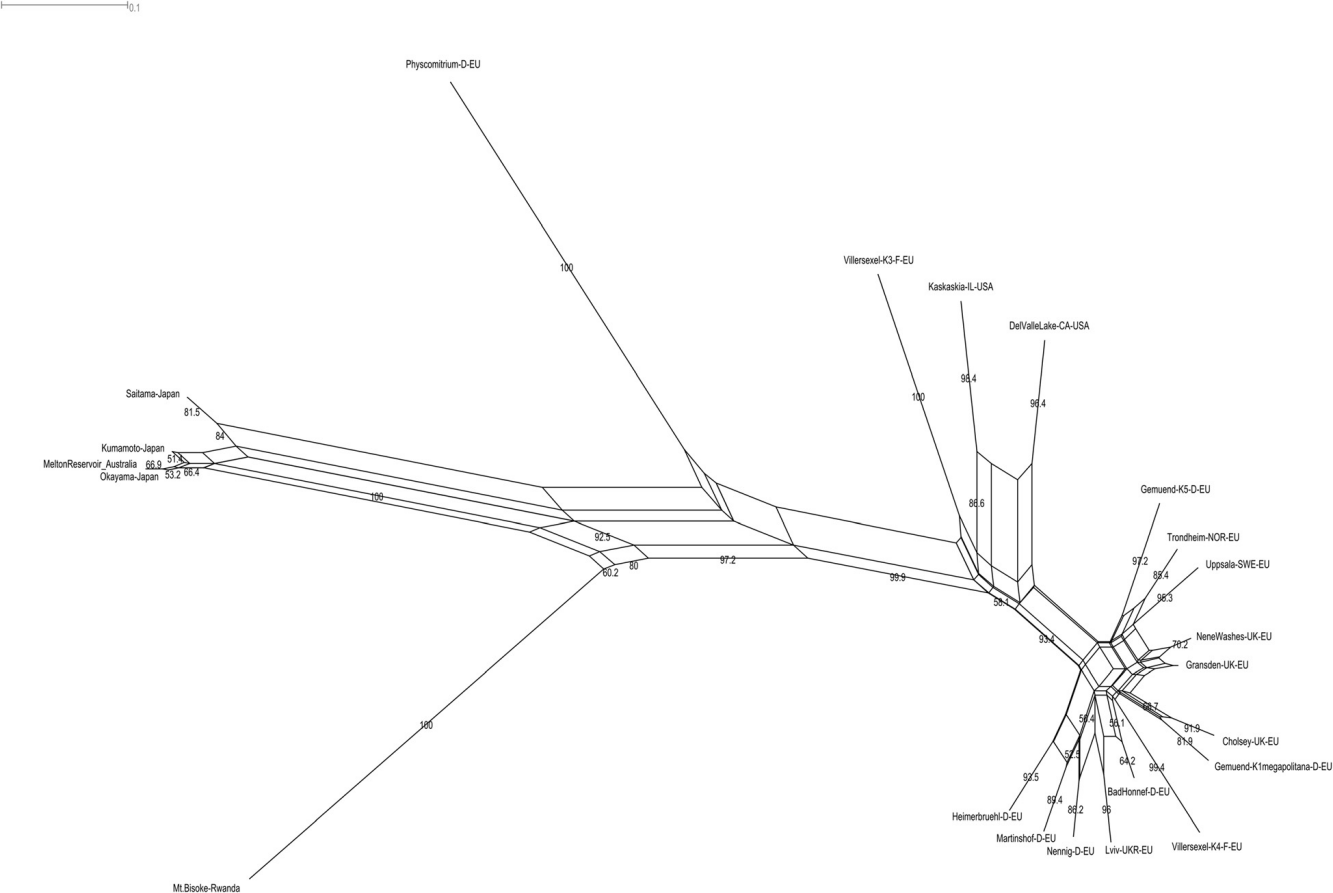
**SSR-based NeighborNet.** Unrooted, equal angled Splitstree NeighborNet based on presence/absence of 170 different sized SSR bands derived from 49 loci. Numbers at the edges represent bootstrap support, shown only for values > 50. Accessions are named with their designation and country.

Sixty-four EST-derived simple sequence repeat (SSR) loci [48] were analyzed, and all *Physcomitrella* accessions (Additional file 2: Table S1) turned out to be of different genotypes as measured by SSRs. In total, 238 alleles were detected (average 3.7 alleles per SSR). The phylogenetic tree based on genetic distances (Figure 5) displays a separation of four major clades. One clearly ramified clade consists of European and North American accessions (*P. patens* ssp. *patens* and *P. patens* ssp. *californica*) with small genetic distances, a second clade is formed by the Japanese and the Australian accessions (*P. patens* ssp. *readeri* and *P. patens* ssp. *californica*), while the African *P. patens* ssp. *magdalenae* is on a separate branch (Figure 5). The genetic distances of the four *Physcomitrella* subspecies clusters to one another are as large as, or even larger than, the distance of *P. sphaericum* to each of them. Large banding size shifts and null alleles obtained in the African and the Japanese-Australian clade point towards sequence changes surpassing the enclosed SSRs and affecting the flanking regions. Visualization of the allele presence/absence data as a network (Figure 6) suggests either sexual recombination and thus long range genetic flow, or the parallel (convergent) evolution of alleles. There are only minor differences between the topologies of Figures 5 and 6. In summary, although some of the nodes are not well supported, the genetic distance data support three independently evolved (polyphyletic) *Physcomitrella* lineages in the same way as the *BRK1* data does.

### The pattern of editing sites supports independent speciation of *Physcomitrella* and hybridization of *Physcomitrium*

**Figure 7:**
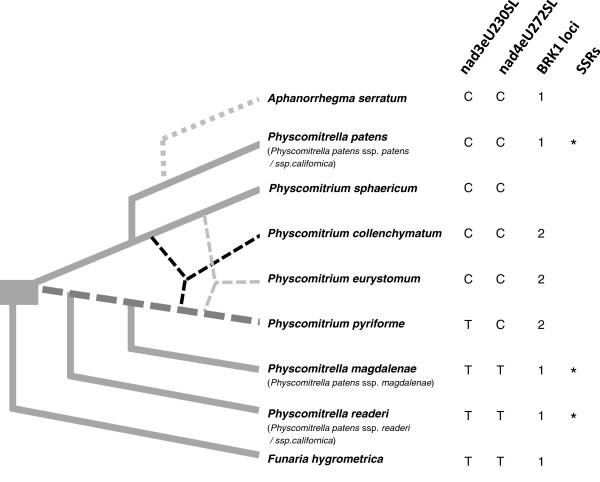
**Overview of molecular features supporting the proposed speciation within the *Physcomitrium-Physcomitrella *species complex.** The re-classified species (subspecies following the former classification of Tan 1979 in brackets) are arranged as supported by data presented in this study, modified after [12]. Dashed lines indicate hybridization events resulting in the three species of *Physcomitrium*. The dotted line illustrates the clustering of *A. serratum* together with *P. patens*. The requirement of editing at particular positions in *nad*3 and *nad*4 is shown. The RNA editing site nomenclature consists of affected gene, position in the reading frame and the resulting amino acid codon change induced by RNA editing [32]. C (Cytidine) at the DNA level indicates an editing site, whereas no editing is required in the case of a thymidine (T) at the appropriate position. The number of *BRK1* loci (homeologs) is depicted for all analyzed species, while *indicates SSR-based support for the three *Physcomitrella* species.

Requirement for RNA editing at eight positions in the mitochondrial genome known to be edited in *P. patens* from Gransden (Europe) was checked for a broad set of accessions across the *Physcomitrium*-*Physcomitrella* species complex (Additional file 2: Table S1, Additional file 7: Table S4). Editing sites in *nad*5, *cox*1 and *ccm*FC, which were conserved between *F. hygrometrica* and *P. patens*, as well as rps14eU137SL (for nomenclature see Figure 7), absent from *F. hygrometrica*[34], were found to be conserved in all investigated species. In contrast, the requirement of editing at positions nad3-230 and nad4-272 varied. While all isolates of *Physcomitrella* from Europe and North America (ssp. *patens* and ssp. *californica*) showed editing at both positions, in accessions of *Physcomitrella* from Australia and Japan (ssp. *readeri* and ssp. *californica*) and *P. patens* ssp. *magdalenae* from Africa a T was already encoded at the appropriate positions, making editing obsolete. This supports the independent speciation of at least two *Physcomitrella* lineages, and the assignment of accessions of *P. patens* ssp. *californica* into different *Physcomitrella* lineages.

Clustering of *A. serratum* to the *P. patens* clade, as shown in the *BRK1*-based phylogeny, was confirmed by the same editing requirements of *A. serratum* and *P. patens* ssp. *patens* in contrast to *Funaria* (Figure 7). All *P. pyriforme* isolates showed a T at position nad3eU230SL and a C at position nad4eU272SL at the DNA level (Figure 7), congruent with *P. pyriforme* being a hybrid species derived from divergent parental lines. The *P. collenchymatum* and *P. eurystomum,* as well as the *Physcomitrium sphaericum* editing sites nad3eU230SL and nad4eU272SL, are akin to the *P. patens* ssp. *patens* clade (Figure 7). The scenario of *P. patens* ssp. *magdalenae* developing from the same parental line as *P. pyriforme* and *P. eurystomum* is further supported by a site to be edited, ccmFCeU52RC, exclusively identified in accessions of these three lineages. An additional putative editing site in *nad*5, nad5eU446SL, is only shared by *P. pyriforme* and *P. patens* ssp. *magdalenae.*

### *In vitro* comparison of gametophytic morphological features

Since sporophytic features are not suitable for distinguishing *Physcomitrella* species, we compared gametophytes grown under identical *in vitro* conditions. The Californian accession (Additional file 8: Figure S4D, Additional file 9: Figure S5D, Additional file 10: Figure S6B) deviates from the European accessions (Additional file 8: Figure S4A-C, Additional file 9: Figure S5A-C, Additional file 10: Figure S6A) by developing much smaller gametophores with smaller leaflets (i.e., non-vascular leaves or phyllids; *cf.* Plant Ontology term PO:0025075). However, consistent with the European accessions, the leaflets of the Californian accession develop a costa in most cases, albeit less pronounced and extending to only three quarters of the leaflets (Additional file 10: Figure S6A, S6B). In the Japanese and the Australian accessions the gametophores and leaflets (Additional file 8: Figures S4E, Additional file 9: Figure S5G-I) are much smaller, typically less than half the size as compared to *P. patens* from Gransden (Europe, Additional file 8: Figure S4A, Additional file 9: Figure S5A, Additional file 10: Figure S6A), comparable to the Californian accession (Additional file 10: Figure S6B, S6C). However, the leaflets do not develop a costa except in very rare cases where a costa may be present, reaching at most half the length of the leaflets (Additional file 10: Figure S6C). The leaflets of the African accession (Additional file 8: Figure S4F, Additional file 9: Figure S5F) are the largest among all analyzed accessions, with up to twice the surface of the European accessions (Additional file 10: Figure S6D). They are orbiculate and much broader than the lanceloate leaflets of the other accessions (Additional file 10: Figures S6D). In summary, leaflet shape and the presence of a costa might be useful to distinguish the *Physcomitrella* accessions, while leaflet/gametophore size seemed rather variable. Of course, this comparison was done only for single accessions from *in vitro* cultivated Funariaceae and one can therefore expect more morphological variance in the field as well as from other accessions grown under *in vitro* conditions.

## Discussion

### Hybridization and polyploidization among the Funariaceae

Here we present data showing that convergent evolution and allopolyploidization are associated with the generation of new species in the Funariaceae. Interspecific hybridization among mosses is an underestimated evolutionary phenomenon [26]. Both, artificial crossings [24,25,27,54] and naturally occurring hybrids have previously been described for the Funariaceae [28-31].

Previously, [12] reported that *Physcomitrium collenchymatum* and *P. eurystomum* are hybrid species, putatively produced from hybridizations between ancestors of modern *P. sphaericum* and *P. pyriforme*. This was based on the observation that these species contained specific polymorphisms (i.e., *sphaericum* and *pyriforme*-like alleles) at four nuclear loci (*adk*, *apr*, *ho1* and *papr*). However, each haploid individual generally contained either the *sphaericum* or *pyriforme*-like allele, but never both. This finding was consistent with either homoploid hybrid speciation, or allopolyploid speciation, followed by a loss of one homeolog at each of these loci (fractionation). The one exception to this pattern was the *ho1* locus where four European isolates of *P. pyriforme* contained two divergent copies of this locus.

Here we found that *P. eurystomum*, *P. collenchymatum*, and five European isolates of *P. pyriforme* have genome sizes larger than those of their putative parent lineages, while they also contain two divergent paralogs of *BRK1*. For these data to be explained by a homoploid hybrid speciation event, we would have to assume both a massive expansion of transposable elements [55] and parallel duplications of the *BRK1* locus. Alternatively, these data might reflect an allopolyploidization event followed by the loss of one paralog at the *adk*, *apr*, (*ho1*) and *papr* loci, a process called fractionation. Our current data do not allow to unambiguously rule out homoploid hybrid speciation, but the frequency of homeolog loss following polyploidy in a wide range of organisms [56] suggests that fractionation may be a more plausible explanation. A comparative analysis of the genome-wide patterns of paralog-loss or retention could potentially provide insights into the genetic interactions among loci or dosage sensitivity.

The evolutionary success of hybrids is also known for seed plants, where allopolyploid offspring are sometimes more successful than the parental lines [21,22]. Contrary to seed plants, which contain alleles in the dominant sporophytic generation, mosses contain only a single gene locus in the dominant haploid generation. Therefore, the homeologs generated through an auto- or allopolyploidization event might represent an additional evolutionary advantage since they represent redundant gene copies (akin to alleles), encoded on haploid segregated chromosomes. In allopolyploid hybrids, gains of genes only encoded by one of the two parental genomes might even be more beneficial if they relay an important advantage that one of the parents did not encode.

In the allopolyploid seed plant cotton the majority of expression biases are thought to be due to sub- and neofunctionalization subsequent to the polyploidization event [57]. Indeed, 60 % of the homeologs are found to be transcriptionally biased, e.g., due to *cis*-regulatory element divergence of the parental genomes [58,59]. Under the conditions applied here, both *BRK1* homeologs are expressed in the same tissue in *P. collenchymatum* and *P. eurystomum,* and in the case of *P. collenchymatum* their expression levels seem not to be heavily biased. Allopolyploid cotton has been estimated to be 1.5 MY old [60], while the separation of the parental lineages within the *Physcomitrium*-*Physcomitrella* species complex occurred ~11 MYA [12]. The hybrids studied here are likely younger than that - but we might also see different patterns or speeds of homeolog divergence between mosses and seed plants in future studies.

With regard to editing site evolution, we consider a T at nad3_230 and nad4_272 to be the ancestral state, since three lineages (*Funaria hygrometrica*, *Physcomitrella readeri* and *Physcomitrella magdalenae*) share this characteristic, while the gain of a C in these positions can be explained by a single mutation per editing site in the lineage giving rise to *Physcomitrium sphaericum* and *Physcomitrella patens* (Figure 7). Under this scenario, editing of nad3eU230SL and nad4eU272SL in the hybrid species *P. eurystomum*, *P. collenchymatum* and *P. pyriforme* might have become feasible by gain (from the parental *P. sphaericum* lineage) of the corresponding nuclear encoded editing factor PPR_56 (Pp1s208_104V6.1, [61]). The gain of the editing requirement (i.e., a C instead of a T in the mitochondrial DNA) at one or both positions in the hybrid species can be explained by either maternal transmission of the organelle and thus transfer of the mutation from the *P. sphaericum* parental lineage, or by the independent gain of mutations (after loss of selection pressure by gain of the nuclear editing factor).

Based on synonymous substitution plots of gene duplication events, a whole-genome duplication in *P. patens* has been hypothesized and dated to ~45 MYA [23]. As this is much later than the Funariidae divergence ~172 MYA [62], and gene family trees including *F. hygrometrica* and *P. patens* usually show clear ortholog pairs [34,63], it is reasonable to assume that the previously shown paleopolyploidization [23] is a common ancestral trait of all Funariaceae. In this light, it is surprising that the *F. hygrometrica* haploid genome size measured here is significantly lower than that of *P. patens*. An explanation for this finding could be a much lower transposon or repetitive element content in *F. hygrometrica*, or a significantly different A/T content. In any case, fractionation (i.e., homeolog loss) apparently occurred to a large extent, since the paleopolyploidization event was not detectable by means of homeologs of *BRK1* in those Funariaceae that are not hybrids.

In summary, (sympatric) speciation following allopolyploidization has apparently occurred several times within the *Physcomitrium*-*Physcomitrella* species complex.

### Revised classification of *Physcomitrella*

By comparing the genetic diversity in the analyzed *Physcomitrella* accessions we found consistency in three main distinctive groupings. One clade includes all European and the two North American accessions (as well as *Aphanorrhegma*), a second clade encompasses the Japanese and the Australian accessions and the third lineage is represented by the African accession. Hence, our results confirm recently published molecular data concerning the *Physcomitrium*-*Physcomitrella* species complex, where an independent origin of three *Physcomitrella* lineages was assumed [11,12]. Since the genetic distances between the three *Physcomitrella* clades are as large as, or even larger than, the genetic distances to *Physcomitrium* species, the three *Physcomitrella* lineages should be regarded as different species. Based on the results presented here, we propose a revised classification, which divides *Physcomitrella* into three species:

1. ***Physcomitrella patens *(Hedw.) Bruch et Schimp.**, Bryol. Eur. 1: 13, 1849. Basionym: *Phascum patens* Hedw. Spec. Musc. 20, 1801.

Synonyms: *Physcomitrium patens* (Hedw.) Mitt., Ann. Mag. Nat. Hist., ser. 2, 8: 363, 1851. *Physcomitrella patens* ssp. *californica* (H.A. Crum & L.E. Anderson) B.C. Tan, J. Hattori Bot. Lab. 46: 334, 1979.

This taxon encompasses the European and North American accessions of *P. patens* ssp. *patens* and the North American accessions of *P. patens* ssp. *californica.*

2. ***Physcomitrella readeri *(Müll. Hal.) I.G. Stone & G.A.M. Scott**, J. Bryol. 7: 604, 1974. Basionym: *Ephemerella readeri* Müll. Hal., Hedwigia 41: 120, 1902.

Synonyms: *Physcomitrium readeri* Müll. Hal., Gen. Musc. Frond. 112. 1900, *nom. inval*., lacking species description. *Physcomitridium readeri* (Müll. Hal.) G. Roth, Außereurop. Laubm. 250, 1911. *Physcomitrella patens* ssp. *readeri* (Müll.Hal.) B.C.Tan, J. Hattori Bot. Lab. 46: 334, 1979.

This taxon encompasses the Australian accessions of *P. patens* ssp. *readeri* and the Japanese accessions of *P. patens* ssp. *californica*.

3. ***Physcomitrella magdalenae *De Sloover,** Bull. Jard. Nat. Belg. 45 : 131, 1975.

Synonyms: *Physcomitrella patens* ssp. *magdalenae* (De Sloover) B.C. Tan, J. Hattori Bot. Lab. 46: 334, 1979. *Aphanorrhegma magdalenae* (De Sloover) Ochyra, Acta Bot. Hung. 29: 178, 1983.

This taxon encompasses the African accessions of *P. patens* ssp. *magdalenae*.

Following this classification, *P. patens*, disjunct in North America and Europe, can be clearly distinguished from *P. readeri* and *P. magdalenae* by distinct gametophytic (but not sporophytic) morphological as well as molecular characteristics.

According to the *BRK1* phylogeny and the pattern of editing sites, the stegocarpous *A. serratum* may also be classified as *Physcomitrella*, although *Physcomitrella* is cleistocarpous. Thus, the Funariaceae type species, *F. hygrometrica*, represents the most highly complex end of a morphological series, with *Physcomitrella*/*Aphanorrhegma* representing the other end. The origin of a cleistocarpous taxon from a stegocarpous taxon is found several times within the acrocarpous mosses. Species with cleistocarpous and stegocarpous members are found e.g. in the genus *Pottia*, where *P. bryoides* and *P. recta* are cleistocarpous while the remaining species are stegocarpous, having either no peristome, or a rudimentary or even well-developed peristome. In conclusion, as previously supposed, sporophytic characteristics cannot be used to resolve the phylogeny of the Funariaceae [11].

Considering an independent evolution for *Physcomitrella* from *Physcomitrium* ancestors [12] and the observed (phylo) genetic distances, the analyzed accessions of the *Physcomitrium-Physcomitrella* species complex may in consequence be classified as a single genus. In this case, *Physcomitrium* would be the correct genus name as it is older than *Physcomitrella*, dating back to 1829. Given our present taxon sampling and data set, *Physcomitrella*, *Physcomitrium* and *Aphanorrhegma* form a species complex that may or may not include additional genera, such as *Entosthodon* and *Bryobeckettia*[11]. Therefore, further phylogenetic studies including more accessions and more genera are required in order to confidently propose a revision in taxonomic classification at the genus level (i.e., uniting the species of the *Physcomitrium*-*Physcomitrella* species complex into a monophyletic genus *Physcomitrium*).

### Disjunct occurrence and long range dispersal of *Physcomitrella*

The habitats of the *Physcomitrella* accessions used in this work show clear similarities, as they grow on moist, often disturbed ground, typically in close proximity to water. *Physcomitrella* shows a cosmopolitan, probably originally holarctic distribution (excluding boreal and tropical regions). While one might expect genetic distances to correlate with geographic distances, the two *P. patens* isolates from Gemünd (Germany) cluster with one of the French Villersexel accessions, “K4” (Gemünd “K5”), and the English Cholsey accession (Gemünd “K1”) respectively; the latter Gemünd accession was characterized as *var. megapolitana* in the field. Also, the accessions from Villersexel (France) cluster in different parts of the *P. patens* genetic distance tree. These genetic distances provide evidence for either high intra-population diversity or long range dispersal.

Moss spores are able to survive in mud for prolonged periods of time, a trait considered important for ephemeral species [64]. In particular, *Physcomitrella* is known to quickly appear on the muddy banks of reservoirs after draining. *P. patens* spores are larger and fewer in number (30 μm diameter, 8,000-16,000 per capsule) than those of *F. hygrometrica* (23 μm, 60,000-170,000) [65], making them a bigger energy reservoir that might be able to boost growth as soon as the conditions are suitable. Spores smaller than 20 μm are easily dispersed by wind [64] and long range dispersal via this mode is evident for *Funaria hygrometrica*[66], also considering its cosmopolitan distribution. Given the larger spore size of *P. patens,* along with the fact that the plants grow on wet soil and have a cleistocarpous capsule, spore dispersal by wind is possible but appears unlikely. Although long distance spore dispersal by wind cannot be excluded for *Physcomitrella*, we suggest that spore distribution by birds [67] along migration routes may also contribute to the observed disjunct distribution patterns. The disjunct distribution of *P. patens* on both sides of the Northern Atlantic may be explained by use of the East Atlantic flyway, while the North American continent is covered by a total of four partially overlapping flyways [68], potentially allowing the spores to spread across the continent. Concerning *P. readeri*, which is found in Japan and Australia, but also in Europe [69], nearly identical ribosomal spacer data between the accessions from England, Australia and Japan suggests long range dispersal. The disjunct habitats in Japan and Australia are covered by the West Pacific as well as by the East Asian-Australasian flyway used by migratory birds [68]. Since the East Asian-Australasian flyway is geographically overlapping with the East Atlantic flyway, dispersal from Japan/Australia to Europe [69] is theoretically possible, although less likely than, e.g., the exchange between Japan and Australia. We hypothesize that *Physcomitrella* might be dispersed via migratory (water) birds along flyways since the observed habitats follow a distribution pattern coinciding with such flyways. When the SSR data are visualized as a network, potential genetic flow is also supported. While reticulate structures are present mainly within *P. patens* and *P. readeri*, there is also evidence for alleles that are potentially exchanged between both species. Interestingly, based on the SSR data no exchange is evident between *P. magdalenae*, *P. sphaericum* and any of the other accessions.

### Independent secondary reduction of Funariaceae sporophyte complexity

Considering the fact that characteristic structural features of moss sporophytes can be correlated with specialized habitats [70], the most likely secondary reduction of the *Physcomitrella* sporophyte to a cleistocarpous capsule with reduced seta and increased spore size may be interpreted as an adaptation to an ephemeral life style. Given the polyphyletic origin of the three cryptic (with regard to sporophytic features) *Physcomitrella* species, a convergent evolutionary process in which the seta is reduced and the capsule is no longer dehiscent can be assumed. *P. patens* inhabits an ephemeral habitat, e.g. on banks of rivers and ponds which dry up in summer or autumn. The species has a short life cycle of up to two months from spore germination to the development of spores [65]. Besides a reduced sporophyte, a shortened life cycle also represents a typical adaptation to a highly unpredictable and ephemeral habitat [70]. In *Physcomitrella*, the gametophore is reduced as well, forming a small rosette with its apparent main function being the production of gametangia and spore capsules. As a semi-aquatic moss, the spores are most likely released into water or mud. This mode of spore dispersal does not require a lid or a peristome at the capsule, but rather the disintegration of the capsule when mature spores have developed.

The Funariaceae feature highly variable sporophyte architectures, and sporophytes of interspecies hybrids usually display intermediate or maternal phenotypes [27]. It has been argued that evolutionary pressure may force changes to moss sporophyte architecture rather than conserving it [11,70]. In summary, we hypothesize that probably parapatric speciation via establishing an ecological niche, namely the resting of spores in the mud, their potential dispersal by birds rather than by wind, and an ephemeral life cycle, has led to the independent evolution of a reduced sporophyte in the three *Physcomitrella* lineages – making them cryptic species if one considers sporophyte morphology alone.

## Conclusions

In this study we present molecular insights into the global genetic diversity of the *Physcomitrium-Physcomitrella* species complex, providing evidence for sympatric speciation involving allopolyploidization, as well as for convergent evolution leading to a reduced sporophyte, large spores, and a colonization of a humid, ephemeral, moist habitat, possibly concomitant with possible parapatric speciation. Primarily, the sequenced *P. patens* isolate from Gransden (Europe) has to date been widely used as an experimental model in comparative plant sciences, followed by the French isolate Villersexel “K3” that has been used for the generation of a genetic map [71] and for crossing experiments with “Gransden” and other isolates [54]. The present collection of axenic *in vitro* cultures of Funariaceae accessions, together with the molecular data presented here, is expected to boost the research into natural variation and trait evolution of this emerging model system representing haploid-dominant land plants. Resequencing of Funariaceae accessions will lead to insights into genome evolution and its coupling to trait evolution.

### Availability of supporting data

The data sets supporting the results of this article are included within the article (and its additional files). *BRK1* sequence data have been submitted to Genbank and are available under the accession numbers KC337119-KC337148.

## Competing interests

The authors declare that they have no competing interests.

## Authors’ contributions

SAR conceived of most of the study. MvS, RR and SAR conceived of the SSR and phenotypic part of the study, MvS performed it. AKB and SAR designed most of the experiments. MSR and SAR designed the editing analyses, MvS the SSR analyses. AKB performed the BRK1 experiments, MSR the editing site analyses, STH the qPCRs/melting studies, MF the flow cytometry/melting studies, SAR the statistics. AKB and SAR performed the phylogenetic analyses. AKB, SMD, BCT and SAR hypothesized on species evolution. AKB and SAR drafted the manuscript, with participation of all authors. All authors read and approved the final manuscript.

## Supplementary Material

Additional file 1: Figure S2 Alignment of BRK1 amino acid sequences. Multiple sequence alignment of BRK1 amino acid sequences from several land plants. The conserved blocks used for primer design are denoted by red boxes. The leading five letters of each sequence identifier denote the species in the common abbreviation (first three letters of the genus, followed by the first two letters of the species, e.g. *PHYscomitrella PAtens*). Click here for file

Additional file 2: Table S1 Funariaceae collection (detailed) and data matrix. Click here for file

Additional file 3: Figure S1 Alignment of genomic *BRK1* sequences. Multiple sequence alignment of amplified and clonal genomic sequences of *BRK1* from different Funariaceae. The exon region is shown in white letters, the intron region in black letters. The species names are sorted alphabetically. Accessions with only one locus of *BRK1* are represented by one sequence of directly sequenced PCR product, whereas two representative clonal sequences are shown for each accession with two loci of *BRK1* (*P. collenchamytum*, *P. eurystomum*, and *P. pyriforme*). Polymorphisms in the exon regions are depicted in red (*P. collenchymatum*) and orange (*P. eurystomum*) boxes. Click here for file

Additional file 4: Table S2 SSR data. Click here for file

Additional file 5: Table S3 Flow cytometric analyses (detailed). Click here for file

Additional file 6: Figure S3 Phylogenetic tree using *BRK1*. Neighbor-joining tree of using *BRK1* (Pp1s35_157V6.1) from selected Funariaceae. The three distinct clades of *Physcomitrella* are highlighted in bold. The distinct loci of *BRK1* for each *Physcomitrium* species are highlighted in boxes. Green boxes: distinct loci of *P. pyriforme* accessions from Europe. Blue boxes: distinct loci of one *P. collenchymatum* accession from North America from two different capsules. Red boxes: distinct loci of *BRK1* from three accessions of *P. eurystomum* from Europe. The numbers at the nodes are derived from 1,000 bootstrap samples. Click here for file

Additional file 7: Table S4 Editing site conservation along the *Physcomitrium-Physcomitrella* species complex. Click here for file

Additional file 8: Figure S4 Habitus of gametophytes. *Physcomitrella* gametophytes grown under standardized *in vitro* conditions on solid mineral medium. **(A)***Physcomitrella patens*, [*Physcomitrella patens* ssp. *patens*] from Gransden, Europe; **(B)***Physcomitrella patens* [*patens* ssp. *patens*] from Lviv, Europe; **(C)***Physcomitrella patens* [*patens* ssp. *patens*] from Illinois, USA; **(D)***Physcomitrella patens* [*patens* ssp. *california*] from California, USA; **(E)***Physcomitrella readeri* [*patens* ssp. *readeri*] from Australia; **(F)***Physcomitrella magdalenae* [*patens* ssp. *magdalenae*] from Rwanda, Africa; **(G)***Physcomitrella readeri* [*patens* ssp. *californica*] from Okayama, Japan; **(H)***Physcomitrella readeri* [*patens* ssp. *californica*] from Kumamoto, Japan; **(I)***Physcomitrella readeri* [*patens* ssp. *californica*] from Saitama, Japan. Click here for file

Additional file 9: Figure S5 Habitus of gametophores and leaflets. *Physcomitrella* accessions grown under standardized *in vitro* conditions on solid mineral medium. (A) *Physcomitrella patens* [*Physcomitrella patens* ssp. *patens*] from Gransden, Europe; (B) *Physcomitrella patens* [*patens* ssp. *patens*] from Lviv, Europe; (C) *Physcomitrella patens* [*patens* ssp. *patens*] from Illinois, USA; (D) *Physcomitrella patens* [*patens* ssp. *california*] from California, USA; (E) *Physcomitrella readeri* [*patens* ssp. *readeri*] Australia; (F) *Physcomitrella magdalenae* [*patens* ssp. *magdalenae*] from Rwanda, Africa; (G) *Physcomitrella readeri* [*patens* ssp. *california*] Okayama, Japan; (H) *Physcomitrella readeri* [*patens* ssp. *californica*] from Kumamoto, Japan; (I) *Physcomitrella readeri* [*patens* ssp. *california*] from Saitama, Japan. Click here for file

Additional file 10: Figure S6 Leaflet details. Leaflets of (A) *Physcomitrella patens* [*Physcomitrella patens* ssp. *patens*] from Gransden, Europe; (B) *Physcomitrella patens* [*patens* ssp. *californica*] from Del Valle Lake, California, USA; (C) *Physcomitrella readeri* [*patens* ssp. *californica*] from Japan, Okayama; (D) *Physcomitrella magdalenae* [*patens* ssp. *magdalenae*] from Rwanda, Africa. Both accessions from Europe and North America reveal a costa, in contrast to those from Okayama, Japan. *Physcomitrella* from Rwanda, Africa has orbiculate leaflets in comparison to the lanceolate leaflets of the other accessions. Click here for file
