# Supplementary material for: Molecular evidence for convergent evolution and allopolyploid speciation within the Physcomitrium-Physcomitrella species complex
Source: BMC Evol Biol. 2014 Jul 11;14:158. doi: 10.1186/1471-2148-14-158 (PMC4227049; doi:10.1186/1471-2148-14-158)
Supplement: Additional file 1: Figure S2 — Alignment of BRK1 amino acid sequences. Multiple sequence alignment of BRK1 amino acid sequences from several land plants. The conserved blocks used for primer design are denoted by red boxes. The leading five letters of each sequence identifier denote the species in the common abbreviation (first three letters of the genus, followed by the first two letters of the species, e.g. PHYscomitrella PAtens). [file 1471-2148-14-158-S1.pdf]

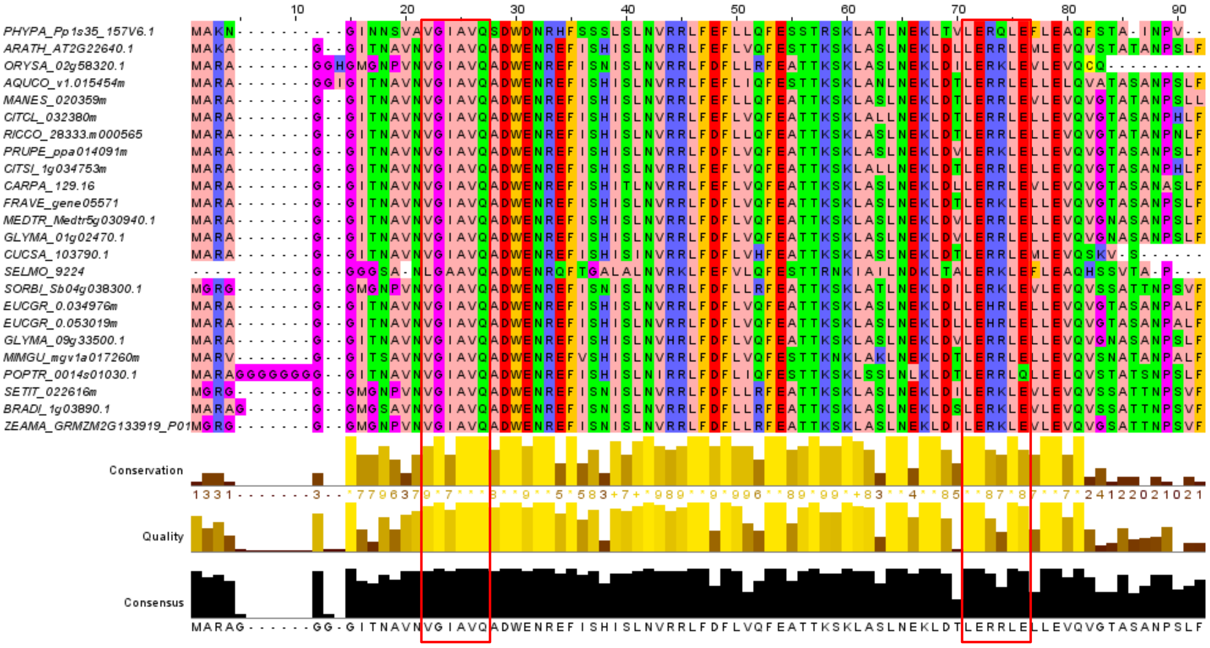

Figure S2 – Alignment of BRK1 amino acid sequences

Multiple sequence alignment of BRK1 amino acid sequences from several land plants. The conserved blocks used for primer design are denoted by red boxes. The leading five letters of each sequence identifier denote the species in the common abbreviation (first three letters of the genus, followed by the first two letters of the species, e.g. *PHYscomitrella PAtens*).
