# Supplementary material for: Molecular evidence for convergent evolution and allopolyploid speciation within the Physcomitrium-Physcomitrella species complex
Source: BMC Evol Biol. 2014 Jul 11;14:158. doi: 10.1186/1471-2148-14-158 (PMC4227049; doi:10.1186/1471-2148-14-158)
Supplement: Additional file 3: Figure S1 — Alignment of genomic BRK1 sequences. Multiple sequence alignment of amplified and clonal genomic sequences of BRK1 from different Funariaceae. The exon region is shown in white letters, the intron region in black letters. The species names are sorted alphabetically. Accessions with only one locus of BRK1 are represented by one sequence of directly sequenced PCR product, whereas two representative clonal sequences are shown for each accession with two loci of BRK1 (P. collenchamytum, P. eurystomum, and P. pyriforme). Polymorphisms in the exon regions are depicted in red (P. collenchymatum) and orange (P. eurystomum) boxes. [file 1471-2148-14-158-S3.pdf]

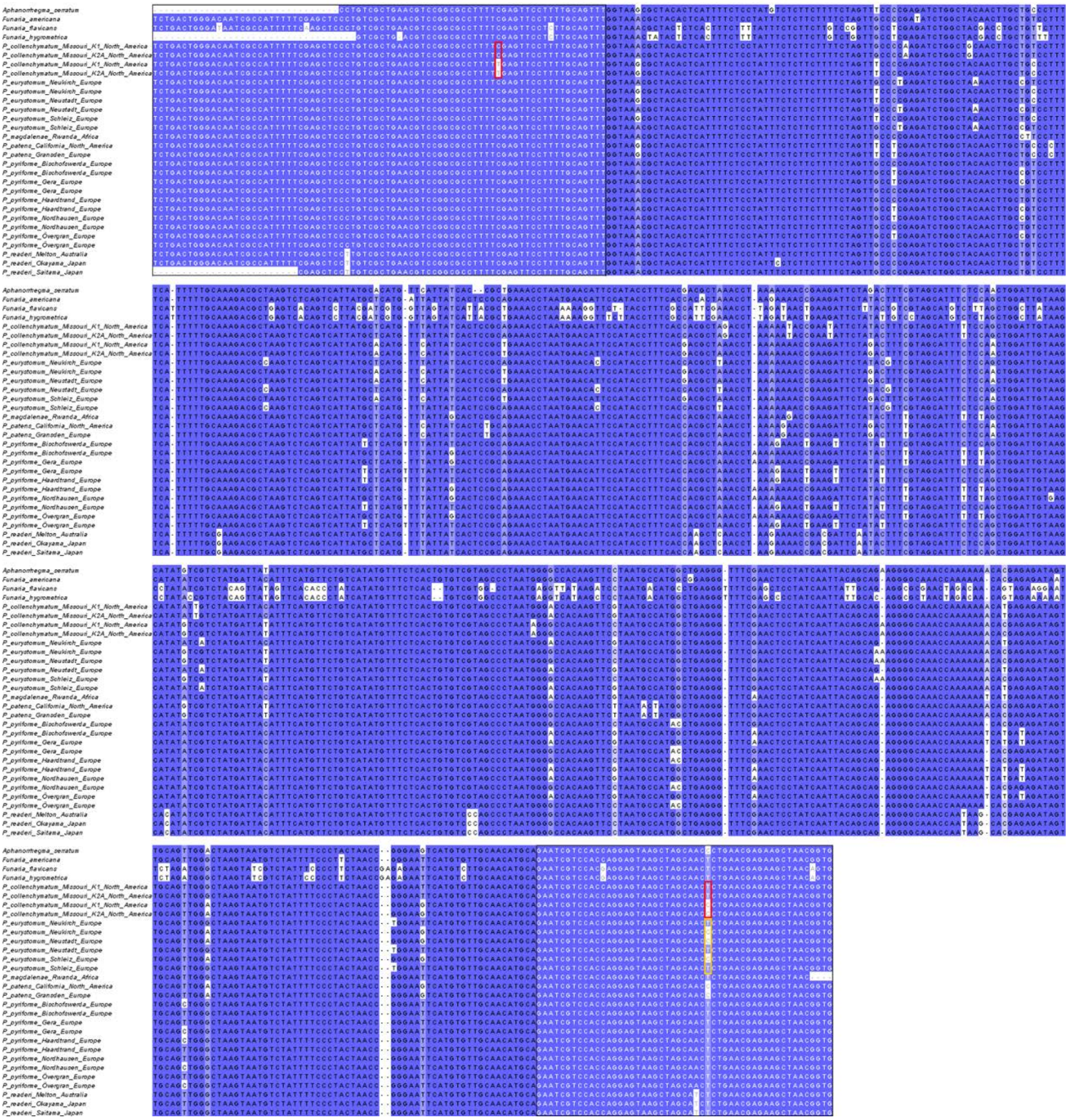

Figure S1 – Alignment of genomic *BRK1* sequences

Multiple sequence alignment of amplified and clonal genomic sequences of *BRK1* from different Funariaceae. The exon region is shown in white letters, the intron region in black letters. The species names are sorted alphabetically. Accessions with only one locus of *BRK1* are represented by one sequence of directly sequenced PCR product, whereas two representative clonal sequences are shown for each accession with two loci of *BRK1* (*P.*

collenchamytum, *P. eurystomum*, and *P. pyriforme*). Polymorphisms in the exon regions are depicted in red (*P. collenchymatum*) and orange (*P. eurystomum*) boxes.
