# Supplementary material for: Molecular evidence for convergent evolution and allopolyploid speciation within the Physcomitrium-Physcomitrella species complex
Source: BMC Evol Biol. 2014 Jul 11;14:158. doi: 10.1186/1471-2148-14-158 (PMC4227049; doi:10.1186/1471-2148-14-158)
Supplement: Additional file 6: Figure S3 — Phylogenetic tree using BRK1. Neighbor-joining tree of using BRK1 (Pp1s35_157V6.1) from selected Funariaceae. The three distinct clades of Physcomitrella are highlighted in bold. The distinct loci of BRK1 for each Physcomitrium species are highlighted in boxes. Green boxes: distinct loci of P. pyriforme accessions from Europe. Blue boxes: distinct loci of one P. collenchymatum accession from North America from two different capsules. Red boxes: distinct loci of BRK1 from three accessions of P. eurystomum from Europe. The numbers at the nodes are derived from 1,000 bootstrap samples. [file 1471-2148-14-158-S6.pdf]

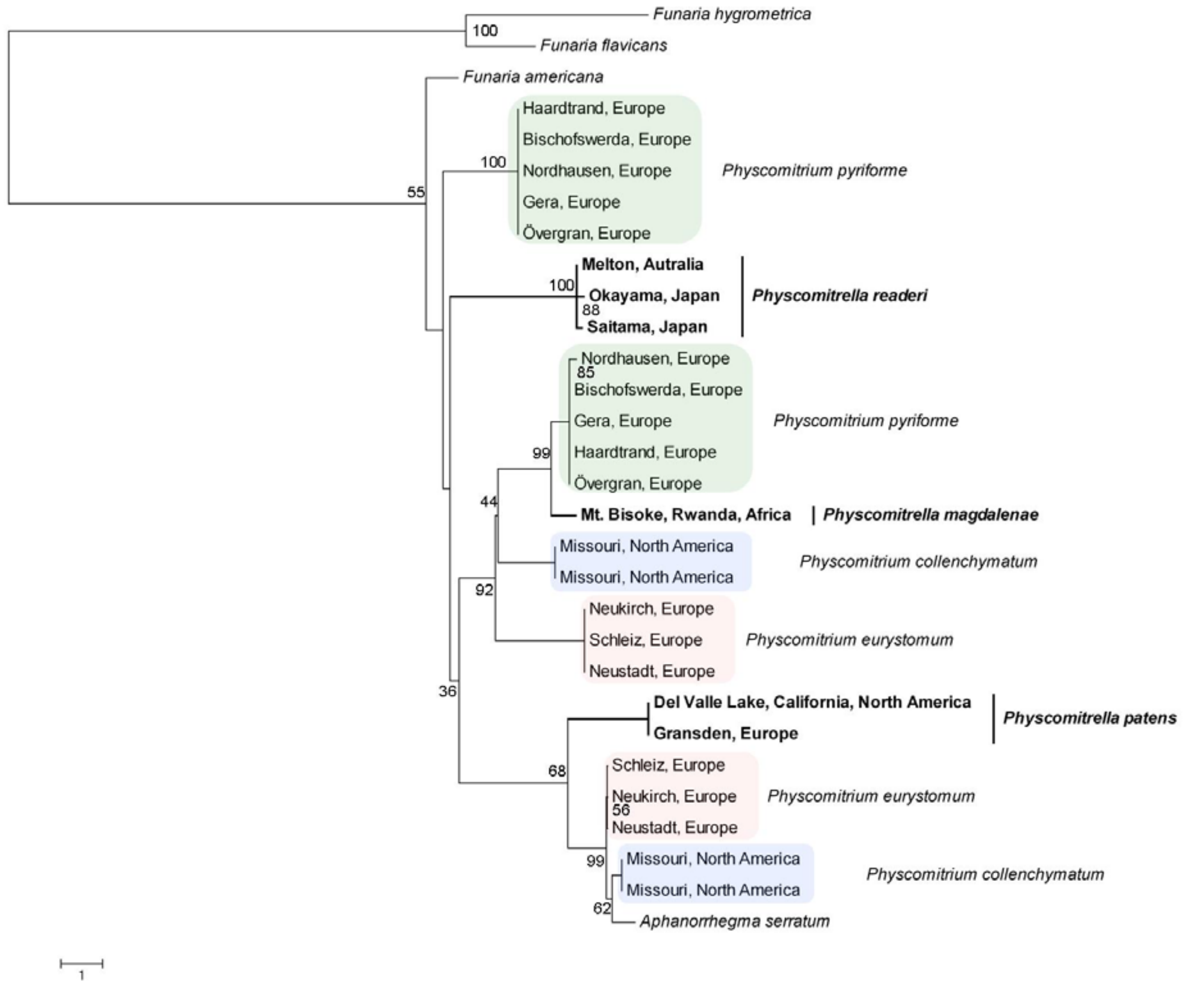

Figure S3 - Phylogenetic tree using *BRK1*

Neighbor-joining tree of using *BRK1* (Pp1s35_157V6.1) from selected Funariaceae. The three distinct clades of *Physcomitrella* are highlighted in bold. The distinct loci of *BRK1* for each *Physcomitrium* species are highlighted in boxes. Green boxes: distinct loci of *P. pyriforme* accessions from Europe. Blue boxes: distinct loci of one *P. collenchymatum* accession from North America from two different capsules. Red boxes: distinct loci of *BRK1* from three accessions of *P. eurystomum* from Europe. The numbers at the nodes are derived from 1,000 bootstrap samples.
