# Supplementary material for: Molecular evidence for convergent evolution and allopolyploid speciation within the Physcomitrium-Physcomitrella species complex
Source: BMC Evol Biol. 2014 Jul 11;14:158. doi: 10.1186/1471-2148-14-158 (PMC4227049; doi:10.1186/1471-2148-14-158)
Supplement: Additional file 8: Figure S4 — Habitus of gametophytes. Physcomitrella gametophytes grown under standardized in vitro conditions on solid mineral medium. (A)Physcomitrella patens, [Physcomitrella patens ssp. patens] from Gransden, Europe; (B)Physcomitrella patens [patens ssp. patens] from Lviv, Europe; (C)Physcomitrella patens [patens ssp. patens] from Illinois, USA; (D)Physcomitrella patens [patens ssp. california] from California, USA; (E)Physcomitrella readeri [patens ssp. readeri] from Australia; (F)Physcomitrella magdalenae [patens ssp. magdalenae] from Rwanda, Africa; (G)Physcomitrella readeri [patens ssp. californica] from Okayama, Japan; (H)Physcomitrella readeri [patens ssp. californica] from Kumamoto, Japan; (I)Physcomitrella readeri [patens ssp. californica] from Saitama, Japan. [file 1471-2148-14-158-S8.pdf]

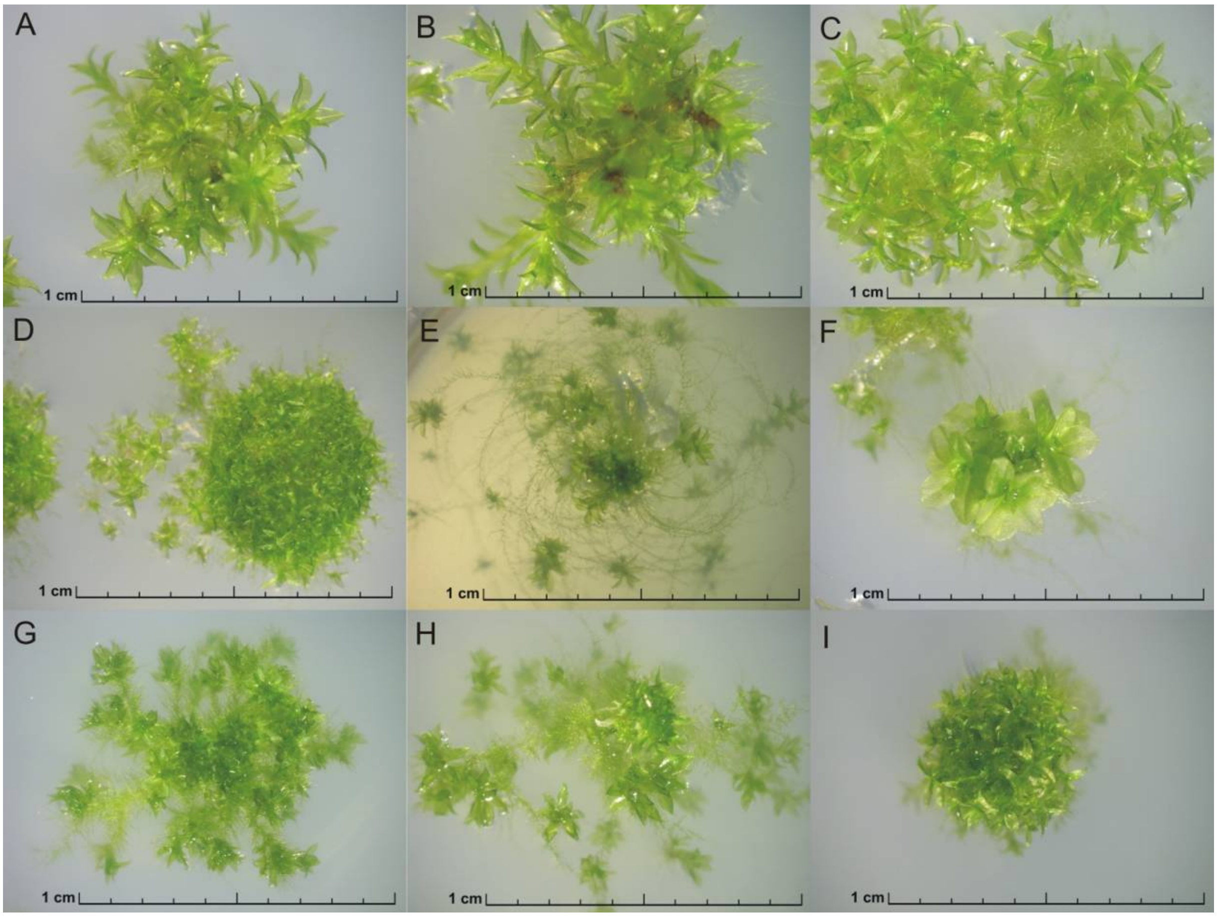

Figure S4 - Habitus of gametophytes

Physcomitrella gametophytes grown under standardized *in vitro* conditions on solid mineral medium. (A) *Physcomitrella patens*, [*Physcomitrella patens* ssp. *patens*] from Gransden, Europe; (B) *Physcomitrella patens* [*patens* ssp. *patens*] from Lviv, Europe; (C) *Physcomitrella patens* [*patens* ssp. *patens*] from Illinois, USA; (D) *Physcomitrella patens* [*patens* ssp. *californi*a] from California, USA; (E) *Physcomitrella readeri* [*patens* ssp. *readeri*] from Australia; (F) *Physcomitrella magdalenae* [*patens* ssp. *magdalenae*] from Rwanda, Africa; (G) *Physcomitrella readeri* [*patens* ssp. *californica*] from Okayama, Japan; (H) *Physcomitrella readeri* [*patens* ssp. *californica*] from Kumamoto, Japan; (I) *Physcomitrella readeri* [*patens* ssp. *californica*] from Saitama, Japan.
