# Supplementary material for: Molecular evidence for convergent evolution and allopolyploid speciation within the Physcomitrium-Physcomitrella species complex
Source: BMC Evol Biol. 2014 Jul 11;14:158. doi: 10.1186/1471-2148-14-158 (PMC4227049; doi:10.1186/1471-2148-14-158)
Supplement: Additional file 9: Figure S5 — Habitus of gametophores and leaflets. Physcomitrella accessions grown under standardized in vitro conditions on solid mineral medium. (A) Physcomitrella patens [Physcomitrella patens ssp. patens] from Gransden, Europe; (B) Physcomitrella patens [patens ssp. patens] from Lviv, Europe; (C) Physcomitrella patens [patens ssp. patens] from Illinois, USA; (D) Physcomitrella patens [patens ssp. california] from California, USA; (E) Physcomitrella readeri [patens ssp. readeri] Australia; (F) Physcomitrella magdalenae [patens ssp. magdalenae] from Rwanda, Africa; (G) Physcomitrella readeri [patens ssp. california] Okayama, Japan; (H) Physcomitrella readeri [patens ssp. californica] from Kumamoto, Japan; (I) Physcomitrella readeri [patens ssp. california] from Saitama, Japan. [file 1471-2148-14-158-S9.pdf]

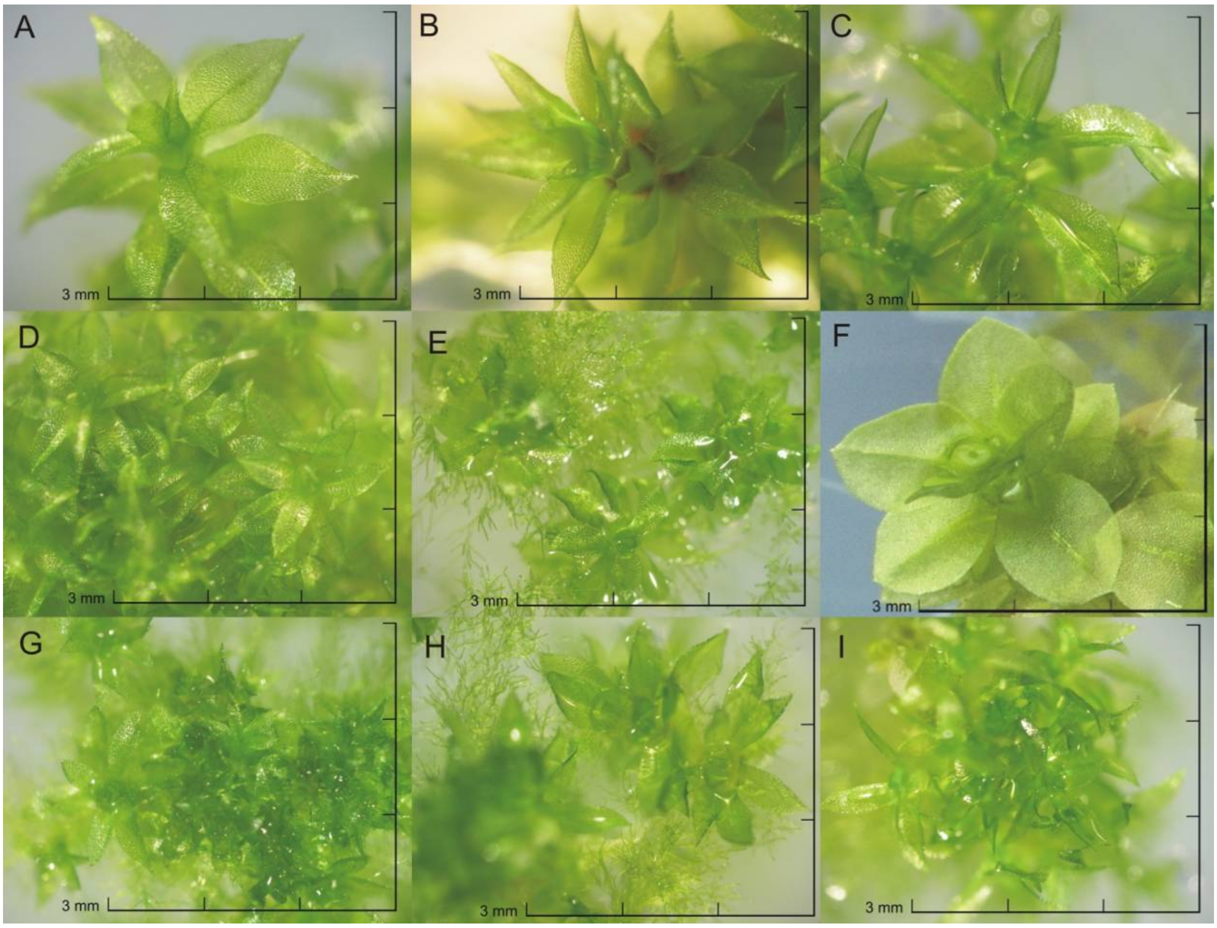

Figure S5 - Habitus of gametophores and leaflets

Physcomitrella accessions grown under standardized *in vitro* conditions on solid mineral medium. (A) *Physcomitrella patens* [*Physcomitrella patens* ssp. *patens*] from Gransden, Europe; (B) *Physcomitrella patens* [*patens* ssp. *patens*] from Lviv, Europe; (C) *Physcomitrella patens* [*patens* ssp. *patens*] from Illinois, USA; (D) *Physcomitrella patens* [*patens* ssp. *california*] from California, USA; (E) *Physcomitrella readeri* [*patens* ssp. *readeri*] Australia; (F) *Physcomitrella magdalenae* [*patens* ssp. *magdalenae*] from Rwanda, Africa; (G) *Physcomitrella readeri* [*patens* ssp. *california*] Okayama, Japan; (H) *Physcomitrella readeri* [*patens* ssp. *californica*] from Kumamoto, Japan; (I) *Physcomitrella readeri* [*patens* ssp. *california*] from Saitama, Japan.
