# Supplementary material for: Molecular evidence for convergent evolution and allopolyploid speciation within the Physcomitrium-Physcomitrella species complex
Source: BMC Evol Biol. 2014 Jul 11;14:158. doi: 10.1186/1471-2148-14-158 (PMC4227049; doi:10.1186/1471-2148-14-158)
Supplement: Additional file 10: Figure S6 — Leaflet details. Leaflets of (A) Physcomitrella patens [Physcomitrella patens ssp. patens] from Gransden, Europe; (B) Physcomitrella patens [patens ssp. californica] from Del Valle Lake, California, USA; (C) Physcomitrella readeri [patens ssp. californica] from Japan, Okayama; (D) Physcomitrella magdalenae [patens ssp. magdalenae] from Rwanda, Africa. Both accessions from Europe and North America reveal a costa, in contrast to those from Okayama, Japan. Physcomitrella from Rwanda, Africa has orbiculate leaflets in comparison to the lanceolate leaflets of the other accessions. [file 1471-2148-14-158-S10.pdf]

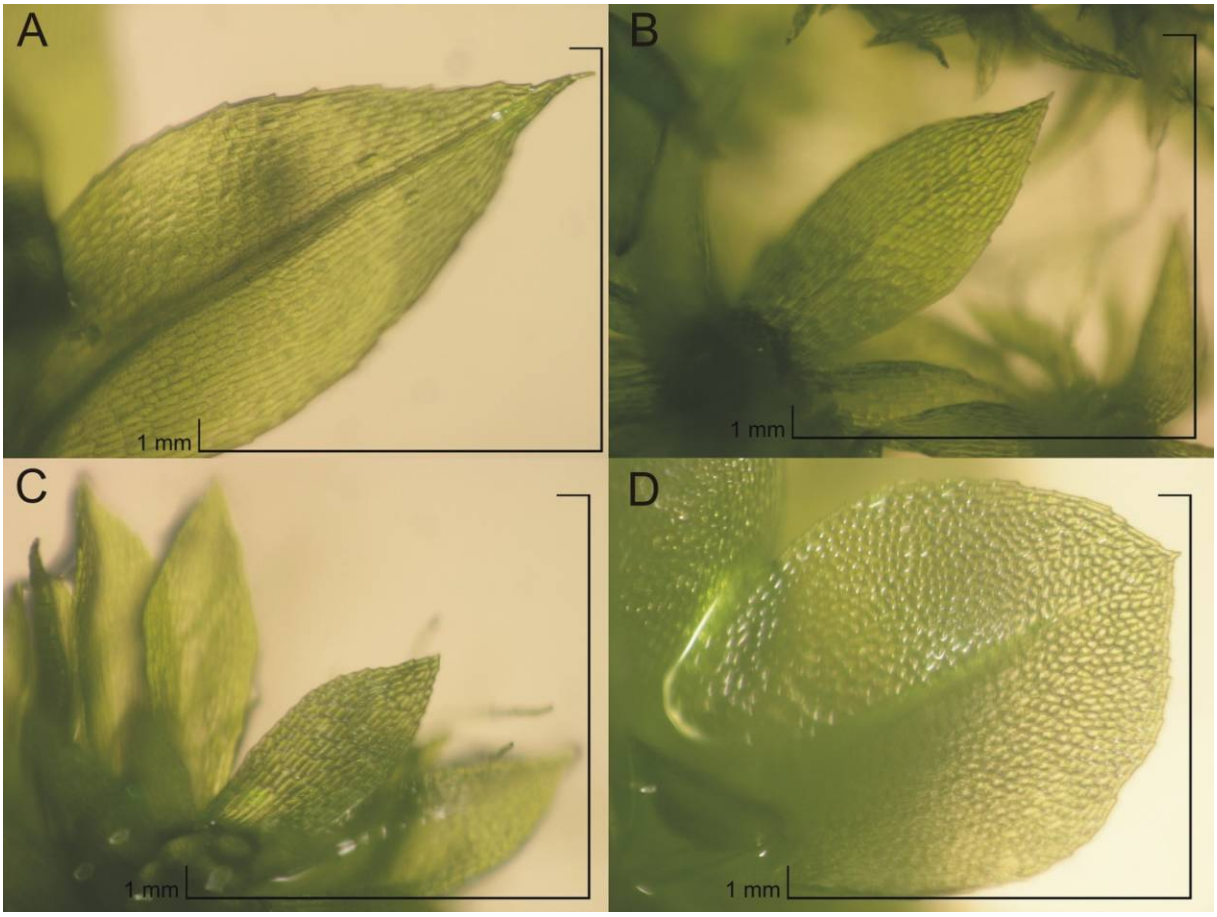

Figure S6 - Leaflet **details**

Leaflets of (A) *Physcomitrella patens* [*Physcomitrella patens* ssp. *patens*] from Gransden, Europe; (B) *Physcomitrella patens* [*patens* ssp. *californica*] from Del Valle Lake, California, USA; (C) *Physcomitrella readeri* [*patens* ssp. *californica*] from Japan, Okayama; (D) *Physcomitrella magdalenae* [*patens* ssp. *magdalenae*] from Rwanda, Africa. Both accessions from Europe and North America reveal a costa, in contrast to those from Okayama, Japan. *Physcomitrella* from Rwanda, Africa has orbiculate leaflets in comparison to the lanceolate leaflets of the other accessions.
